# A mendelian randomization study with populations of European ancestry rules out a causal relationship between inflammatory bowel disease and colorectal cancer

**DOI:** 10.3389/fgene.2022.949325

**Published:** 2022-08-24

**Authors:** Fan Li, Yuyuan Liu, Zhaodi Wang, Qi Zhao, Yuqin Li, Tongyu Tang

**Affiliations:** ^1^ Department of Gastroenterology, The First Hospital of Jilin University, Changchun, China; ^2^ Norman Bethune Health Science Center, Jilin University, Changchun, China

**Keywords:** inflammatory bowel disease, ulcerative colitis, crohn’s disease, colorectal cancer, mendelian randomization

## Abstract

**Background:** Ulcerative colitis (UC), a subtype of inflammatory bowel disease (IBD), has been found to be associated with colorectal cancer (CRC) in observational studies, but there is no evidence to support a causal relationship or reverse causality between the two diseases.

**Methods:** We employed two-sample bidirectional Mendelian randomization to estimate an unconfounded bidirectional causal relationship between IBD (including UC and Crohn’s disease (CD)) and colorectal cancer. After searching IEU GWAS database and filtering SNPs, we applied a variety of MR methods including IVW method using qualified instrumental variables, and conducted sensitivity analysis to detect the heterogeneity and pleiotropy of instrumental variables.

**Results:** After using three groups of SNPs (CD: 106, UC: 113, IBD: 70), the IVW method MR analysis showed that the results were not significant (result for UC: odds ratio (OR) [95% Confidence Interval (CI)]: 0.9998 [0.9991–1.0005], p value: 0.58; result for CD: OR [95%CI]: 0.99962 [0.99912–1.00012], p value: 0.14; results for IBD: OR [95%CI]: 0.99959 [0.99869–1.00048], p value: 0.36). MR-Egger regression, WM method and MR-RAPS method reached the same conclusion. Sensitivity analysis did not reveal heterogeneity and pleiotropy. Bidirectional MR analysis was performed using the same procedure, and the results of IVW MR analysis were also not significant (result for CD: OR [95%CI]: 1.07985 [0.00049–2372.38304], p value 0.98; result for UC: OR [95%CI]: 0.27117 [0.00014–528.3707], p value: 0.74; result for IBD: OR [95%CI]: 0.47101 [0.0001–2242.94159], p value: 0.86). MR-Egger regression, WM method and MR-RAPS method also reached the same conclusion. Sensitivity analysis did not find any evidence of heterogeneity and pleiotropy.

**Conclusion:** Contrary to the conclusions of previous observational studies, a two-sample MR analysis did not find a causal relationship or reverse causal relationship between IBD and CRC. Sporadic CRC (sCRC) may differ in pathogenesis from IBD-related CRC.

## Introduction

Inflammatory bowel disease (IBD) is a chronic, relapsing autoimmune condition that can be manifested in two forms: Crohn’s disease (CD) and ulcerative colitis (UC). The incidence and prevalence of IBD has increased over time in different regions of the world, exceeding 0.3% in many countries and indicating that it has become a global problem (Ng et al., 2017). Colorectal cancer is the second most common cancer in women and the third most common in men (Dekker et al., 2019), responsible for nearly 700,000 deaths each year (Brody, 2015). With increasing colonoscopy screening, its incidence levels in developed countries have plateaued (Dekker et al., 2019). Risk factors for colorectal cancer include age, dietary habits, obesity, smoking and lack of physical activity (Dekker et al., 2019).

It has been found that patients with IBD are at an increased risk of colorectal cancer (called colitis-associated colorectal cancer, CAC, or IBD-related CRC). The first large meta-analysis, conducted in 2001 by Eaden and co-authors, assessed the CRC risk in IBD patients and showed a risk of 2% at 10 years after UC diagnosis, 8% at 20 years, 18% at 30 years, and 3.7% for an overall CRC prevalence (Eaden et al., 2001).

The majority of published studies on the relationship between inflammatory bowel disease and colorectal cancer are observational. Since traditional observational finding can be easily affected by underlying confounding factors such as dietary habits and age and reverse causation, a new approach to causality is needed.

By using genetic variants as proxies for increased or decreased exposure to a risk factor, Mendelian randomization (MR) is a good approach to detect the effect of exposure on disease onset (Smith and Ebrahim, 2003; Emdin et al., 2017). When randomly assigned, genetic variants follow Mendel’s laws. Considering that these genetic variants are associated with exposure, MR analysis is a method of natural randomization and is not subject to confounding factors (Emdin et al., 2017). Mendelian randomization studies are, therefore, more convincing and reliable than traditional observational studies (Yang et al., 2021). They are widely used in the detection of causality in the onset of disease. Some of MR studies have even overturned our common beliefs, as the MR conclusion of HDL-cholesterol being not a protective factor for coronary heart disease did in the study conducted by Holmes et al. (Holmes et al., 2015).

In this study, SNPs related to risk factors were used as instrumental variables to conduct two-way MR analysis between IBD and CRC, in an attempt to uncover the impact of IBD on colorectal cancer and provide new insights for the prevention of CRC in IBD patients and the pathogenesis of IBD-related CRC.

## Methods

### Data source

We selected summary-level datasets from IEU GWAS MR-base database (Hemani et al., 2018). The database contains 227,808,842,007 genetic associations from 40,027 GWAS summary-level datasets for search or download.

Genetic variants associated with IBD were derived from a trans-descent genome-wide association studies (GWAS) by the international IBD Genetics Consortium (IIBDGC) (Liu et al., 2015). Diagnosis of IBD in these studies was based on imaging, endoscopic and histopathological evaluations. The GWAS IDs of these datasets are “ieu-a-12″, “ieu-a-294″, “ieu-a-970".

We used “Colorectal cancer” as a keyword to search on the IEU GWAS website and selected the GWAS summary data with ID “ieu-b-4965″ as the genetic variation associated with colorectal cancer. Data from this GWAS study were obtained from the United Kingdom Biobank (Sudlow et al., 2015) and included 5,657 colorectal cancer patients and 377,673 controls. A total of 11,738,639 SNPs were available for analysis. This dataset includes patients diagnosed with colorectal cancers of different sites including: C18, C19, andC20, according to ICD10 disease codes (https://data.bris.ac.uk/data/dataset/aed0u12w0ede20olb0m77p4b9). The exposure and outcome data was given in per SD unit.

Population stratification is a common source of bias in MR studies. The allele frequencies of the same SNP may differ between populations of different ancestry, which may be associated with certain risk factors (Emdin et al., 2017; Larsson et al., 2019). Therefore, in order to reduce population stratification, all individuals included in this MR study were of European origin. Table 1 showed a summary of the study population, the number of genetic variants (i.e. single nucleotide polymorphisms (SNPs)).

**TABLE 1:** Characteristics of the study population.

Phenotype	SNPs available	Case	Sample size	Author	Year published	Population
IBD	157,116	31,665	65,642	Liu et al	2015	European
CD	124,888	17,897	51,874	Liu et al	2015	European
UC	156,116	13,768	47,745	Liu et al	2015	European
Colorectal cancer	11,738,639	5,657	377,673	Burrows et al	2021	European

### IV selection

Based on the GWAS summary data selected above, we developed a series of criteria to filter SNP. We selected SNPs associated with risk factors at the genome-wide significant level (*p* value less than 5 × 10^–8^), and removed SNPs with linkage disequilibrium (LD) based on *r* ^2^ < 0.01, window size >5,000 kb. The correlation data of SNPs associated with risk factors were extracted from the outcome summary dataset, and the SNPs missing in the outcome dataset were replaced by proxy SNPs (if any) with high LD in European populations. In this study, ambiguous SNPs and palindromic SNPs were removed.

We calculated the F-statistic for each SNP to measure its strength as IV. The F statistic functions as a measure of strength of the instrumental variable SNP to explain the risk factor. F-statistics of SNPs <10 are considered weak instrumental variables (Burgess et al., 2017) and were excluded.

MR-PRESSO test was performed to identify and exclude SNPs with potential pleiotropy. We applied MR-Steiger test to detect the direction of causal estimates for each SNP, and SNPs with the wrong direction were eliminated. Finally, we used PhenoScanner (Staley et al., 2016) to test each SNP for possible associations with confounders, and those SNPs that might violate the independence assumption were removed. After several strict filtering, the remained SNPs were considered to be eligible instrumental variables.

### Research design

To perform MR analysis, the instrumental variables must satisfy the assumptions listed below. Firstly, instrumental variables are significantly correlated with risk factors (relevance assumption). Secondly, instrumental variables are not related to any confounding factor (independence assumption). Thirdly, instrumental variables only indirectly affect outcomes through risk factors (exclusion restriction assumption).

**FIGURE 1:**
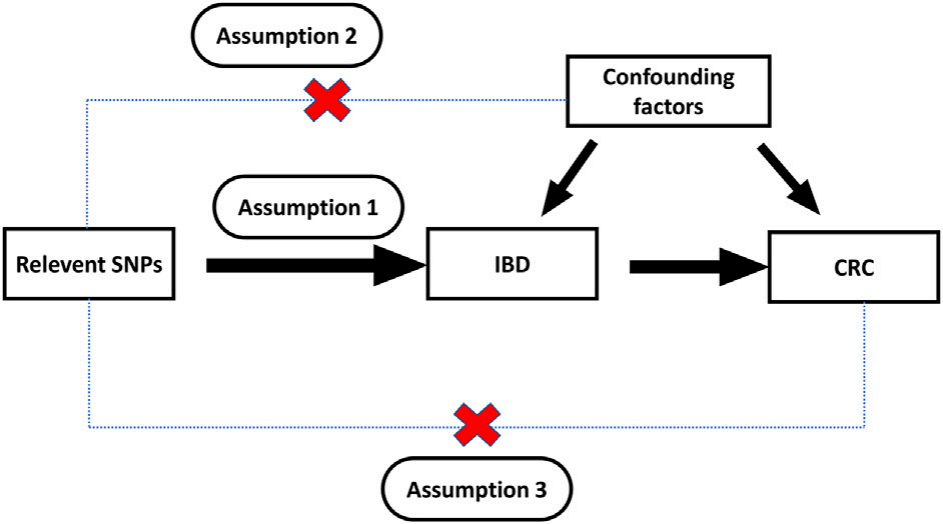
Directed acyclic graph composed of the genetic instrument, exposure, and outcome.

We constructed a directed acyclic graph in Figure 1 using instrumental variables (SNPs), risk factors (CD, UC, IBD) and outcomes (colorectal cancer) to illustrate the basic assumptions of MR study. Based on different assumptions, we applied several robust MR methods to calculate estimates of the effect of IBD on CRC: inverse variance weighted (IVW), MR-Egger regression, weighted median (WM), and MR-robust adjusted profile score (MR-RAPS).

The IVW method, the most commonly used method for MR analysis, use meta-analysis approach to combine ratio estimates of SNPs in an inverse variance weighted way and obtain an estimate of the effect of risk factors on outcomes (Burgess et al., 2013). Ratio estimates are the ratio of the effect of a single SNP on the outcome divided by the effect on the risk factor (with all associations assumed to be log-linear) (Thomas and Conti, 2004). MR-Egger regression is similar to the IVW method, but the intercept term of MR-Egger can assess horizontal pleiotropy (Burgess and Thompson, 2017). The MR-Egger regression is preferred when there is evidence of pleiotropy. The MR-Egger method is based on the NO Measurement Error (NOME) assumption. We also calculated I^2^ values to quantify the extent to which the NOME assumption was violated by MR-Egger. The results should be corrected when I^2^<90% (Bowden et al., 2016b). The WM method can produce correct estimates when less than half of the instrumental variables are invalid (Bowden et al., 2016a; Burgess et al., 2019).

In addition, this study also used the newly developed MR-RAPS method. Due to the use of random effects distribution to simulate the pleiotropic effects of genetic variation, MR-RAPS method is more robust than traditional MR methods (Zhao et al., 2020).

The statistical power of the study was calculated using a web power calculator (https://sb452.shinyapps.io/power/) (Burgess, 2014). The final result was statistically significant when *p* value <0.05.

In a part of meta-analysis involved in this study, we applied the Cochrane Q test and calculated I^2^ to estimate the heterogeneity. The heterogeneity was considered significant when I^2^ was <40% or Q-P value < 0.05. In such cases, a random effects model was used (Bowden et al., 2017).

We apply MR-Steiger to validate the overall direction of causal estimation to ensure robust results (Hemani et al., 2017). We also sought to explore the causal effect of colorectal cancer on the pathogenesis of IBD. The contributions of the datasets therefore were interchanged for bidirectional MR analysis. We extracted significant and independent SNPs without linkage disequilibrium from the dataset “ieu-b-4965ˮ, and then extracted outcome information of those SNPs in the GWAS summary data of IIBDGC (Liu et al., 2015; Hemani et al., 2018). The MR analysis was then performed using the aforementioned MR methods.

In order to address the problem of multiple testing, the results of MR analysis and sensitivity analysis of effect of colorectal cancer on IBD were considered to be statistically significant when P was below 0.0167 (i.e. 0.05/3).

All statistical analyses in this study were performed using R (version 4.1.2) and R packages “TwoSampleMR,” “MRPRESSO,” “mr.raps".

### Data visualization

Scatter plots of the effect of each SNP on risk factors and outcomes and regression curves for causal estimates were made in this study. Plots were made based on the results of leave-one-out tests. Forest plots was drawn based on the results of the final causal estimation. The experimental bias was shown by funnel plots (Egger et al., 1997).

## Results

### Filter instrument variables

A total of 167 SNPs related to CD, 183 related to UC, and 119 related to IBD were selected at the genome-wide significance level. In the colorectal cancer dataset, we selected 12 CD-related, eight UC-related, and 12 IBD-related proxy SNPs, and due to the lack of SNP data, we removed 2 CD-related, five UC-related, and two IBD-related SNPs.

No weak instrumental variables were found, and all F statistics were greater than 10 (ranges of F statistics: 29.9–724.5 (CD), 30.1–320.3 (UC), 29.8–187.2 (IBD)). The mean values of the F-statistics were 78.8 (CD), 69.7 (UC), 53.5 (IBD).

The results show that the selected SNPs meet the relevance assumption of MR study. We found and removed 10 (CD), 13 (UC), 5 (IBD) palindromic SNPs and 1(CD), 0(UC), 2(IBD) ambiguous SNPs, with several SNPs being both palindromic and ambiguous. Leave-one-out analysis (Supplementary Figures S1–S3) did not find SNPs that significantly affected the outcome.

Pleiotropy analysis was performed with MR-PRESSO test. Using PhenoScanner, 48 (CD), 52 (UC), 41 (IBD) SNPs were manually removed. PhenoScanner was used to find other traits associated with SNPs. We removed certain SNPs associated with confounders, such as glycosylated hemoglobin (Xu et al., 2016), triglycerides (Tian et al., 2015; O'Sullivan et al., 2022), BMI (Johnson et al., 2013), etc., which are known for horizontal pleiotropy, could influence the occurrence of colorectal cancer through other pathways, and violated the exclusion restriction assumption of MR study. One SNP in the wrong causal direction associated with IBD was removed by MR-Steiger analysis. The filtering of SNPs is shown in Table 2.

**TABLE 2:** Numbers of Excluded SNPs and identified instrumental SNPs.

Exposures	Outcomes	Miss in outcome	Weak IV	Pleiotropy	Leave-one out	Palindromic structure	Ambiguous SNP	Wrong casual direction	Eligible SNP
CD	**CRC**	2	0	48	0	10	1	0	106
UC	**CRC**	5	0	52	0	13	0	0	113
IBD	**CRC**	2	0	41	0	5	2	1	70
CRC	**CD**	5	0	0	0	0	0	0	4
CRC	**UC**	5	0	0	0	0	0	0	4
CRC	**IBD**	5	0	0	0	0	0	0	4

Finally, after rigorous screening, there were 106 (CD), 113 (UC), 70 (IBD) SNPs left as qualified instrumental variables for the MR analysis. These instrumental variables respectively explained 10.1, 38.1, and 27.6% of the variance of CD, UC and IBD.

### Main results

Tables 3, 4 showed the causal estimates of all MR analysis and sensitivity analyses.

**TABLE 3:** The effect estimates of MR analyses.

Risk factors	Outcomes	MR methods	OR	95% CI lower	95% CI upper	*p* Value
CD	**CRC**	MR Egger	1.0001851	0.998845	1.001527	0.78
		Inverse variance weighted (multiplicative random effects)	0.9996213	0.9991	1.00014	0.15
		Inverse variance weighted (fixed effects)	0.9996213	0.99912	1.00012	0.14
		Weighted median	0.9998874	0.99905	1.00072	0.79
		MR RAPS	0.9996161	0.99911	1.00012	0.14
UC	**CRC**	MR Egger	0.99984	0.997934	1.00175	0.87
		Inverse variance weighted (multiplicative random effects)	0.9998	0.9991	1.0005	0.58
		Inverse variance weighted (fixed effects)	0.9998	0.99918	1.00042	0.53
		Weighted median	0.9998061	0.9988	1.00081	0.71
		MR RAPS	0.9997962	0.99916	1.00043	0.53
IBD	**CRC**	MR Egger	1.0001262	0.997433	1.002827	0.93
		Inverse variance weighted (multiplicative random effects)	0.999585	0.99869	1.00048	0.36
		Inverse variance weighted (fixed effects)	0.999585	0.99887	1.0003	0.26
		Weighted median	0.9996918	0.99854	1.00085	0.60
		MR RAPS	0.9995729	0.99885	1.0003	0.25
CRC	**CD**	MR Egger	5.45E-23	7.86E-55	3.79E+09	0.16
		Inverse variance weighted (multiplicative random effects)	1.0798477	0.00049	2372.38304	0.98
		Inverse variance weighted (fixed effects)	1.0798477	0.00403	289.31816	0.98
		Weighted median	0.1966456	0.00019	200.11424	0.65
		MR RAPS	1.081815	0.00375	312.40734	0.98
CRC	**UC**	MR Egger	4.32E-20	2.74E-51	6.81E+11	0.19
		Inverse variance weighted (multiplicative random effects)	0.2711656	0.00014	528.3707	0.74
		Inverse variance weighted (fixed effects)	0.2711656	0.00267	27.49149	0.58
		Weighted median	0.0233731	0.00007	8.20181	0.21
		MR RAPS	0.259751	0.00245	27.49431	0.57
CRC	**IBD**	MR Egger	3.24E-21	9.76E-59	1.07E+17	0.22
		Inverse variance weighted (multiplicative random effects)	0.4710136	0.0001	2242.94159	0.86
		Inverse variance weighted (fixed effects)	0.4710136	0.00146	152.34891	0.80
		Weighted median	0.0582004	0.00004	95.9504	0.45
		MR RAPS	0.4617744	0.00133	160.20998	0.80

**TABLE 4:** Tests of MR-Steiger casual direction, MR-Egger I^2^, heterogeneity and pleiotropy.

Risk factors	Outcomes	Heterogeneity test				MR-Egger interception	*p* Value	MR-steiger casual direction	MR-egger I^2^
		MR methods	Q	*p* Value	I^2^				
CD	**CRC**	MR-Egger	112.7	0.26	0.08	-7.74E-05	0.37	TRUE	0.99
		Inverse variance weighted	113.6	0.27	0.08				
UC	**CRC**	MR-Egger	141.8	0.03	0.22	-4.17E-06	0.96	TRUE	0.99
		Inverse variance weighted	141.8	0.03	0.21				
IBD	**CRC**	MR-Egger	105.9	0.00	0.36	-6.02E-05	0.67	TRUE	0.98
		Inverse variance weighted	106.2	0.00	0.35				
CRC	**CD**	MR-Egger	0.6	0.73	0.00	0.13	0.15	TRUE	0.97
		Inverse variance weighted	5.7	0.13	0.47				
CRC	**UC**	MR-Egger	2.8	0.24	0.29	0.11	0.19	TRUE	0.97
		Inverse variance weighted	8.1	0.04	0.63				
CRC	**IBD**	MR-Egger	2.6	0.27	0.23	0.12	0.23	TRUE	0.97
		Inverse variance weighted	6.4	0.09	0.53				

According to the IVW analysis, there was no causal relationship between the incidence of IBD and the occurrence of colorectal cancer, and no obvious heterogeneity was found between the SNPs in group of CD on CRC. Because of heterogeneity, the IVW multiplicative random effects model was applied in other two groups (results for CD: OR [95%CI]: 0.99962 [0.99912–1.00012], *p* value: 0.14, Q_*p* value: 0.27, I^2^: 0.08; results for UC: OR [95%CI]: 0.9998 [0.9991–1.0005], *p* value: 0.58, Q_*p* value: 0.03, I^2^: 0.21; results for IBD: OR [95%CI]: 0.99959 [0.99869–1.00048], *p* value: 0.36, Q_*p* value: 0.003, I^2^: 0.35).

We calculated the intercept term of the MR-Egger regression and found no evidence of horizontal pleiotropy (result for CD: MR-Egger interception: 7.4?10^–5^, *p* value: 0.37; result for UC: MR-Egger interception: 4.17?10^–6^, *p* value: 0.96; result of CD: MR-Egger interception: 6.02?10^–5^, *p* value: 0.67).

The MR-Egger, WM and MR-RAPS methods also suggest that the incidence of IBD has no causal relationship with the occurrence of colorectal cancer.

The leave-one-out analysis (Supplementary Figures S1–S3) showed that the results are robust. MR-Steiger test confirmed that the casual direction of each MR study was correct. The evidence of horizontal pleiotropy and outliers were not found by the MR-PRESSO test. The statistical power of this study is relatively low (CD: 0.05, UC: 0.05, IBD: 0.05).

### Bidirectional MR analysis

To explore the causal effect of CD, UC and IBD on colorectal cancer, we extracted four SNPs separately as significant and independent instrumental variables for colorectal cancer. The four instrumental variables explained 1.78% of the variance of the risk factors. After calculation, it was found that there were no weak instrumental variables, and all F statistics were greater than 10 (range 35.1–95.8, mean 61.0).

The IVW method analysis showed that the incidence of colorectal cancer was not causally related to the occurrence of IBD. Because of heterogeneity, the IVW multiplicative random effects model was applied. Results for CD: OR [95%CI]: 1.07985 [0.00049–2372.38304], *p* value: 0.98, Q_*p* value: 0.13, I^2^: 0.47; results for UC: OR [95%CI]: 0.27117 [0.00014–528.3707], *p* value: 0.74, Q_*p* value: 0.04, I^2^: 0.63; results for IBD: OR [95%CI]: 0.47101 [0.0001–2242.94159], *p* value: 0.86, Q_*p* value: 0.09, I^2^: 0.53.

We calculated the intercept term of the MR-Egger regression and found no evidence of horizontal pleiotropy (result for CD: MR-Egger interception: 0.13, *p* value: 0.15; result for UC: MR-Egger interception: 0.11, *p* value: 0.19; result for CD: MR-Egger interception: 0.12, *p* value: 0.23).

The leave-one-out analysis (Supplementary Figures S4–S6) showed that the results are robust. MR-Steiger test confirmed that the casual direction of each MR study was correct. The evidence of horizontal pleiotropy and outliers were not found by the MR-PRESSO test. The statistical power is 0.20 (CRC on CD), 1.00 (CRC on UC), 1.00 (CRC on IBD).

In conclusion, we did not find a causal relationship between the incidence of IBD and the incidence of colorectal cancer, nor did we find a causal relationship between the incidence of colorectal cancer and the incidence of IBD.

## Results visualization

**FIGURE 2:**
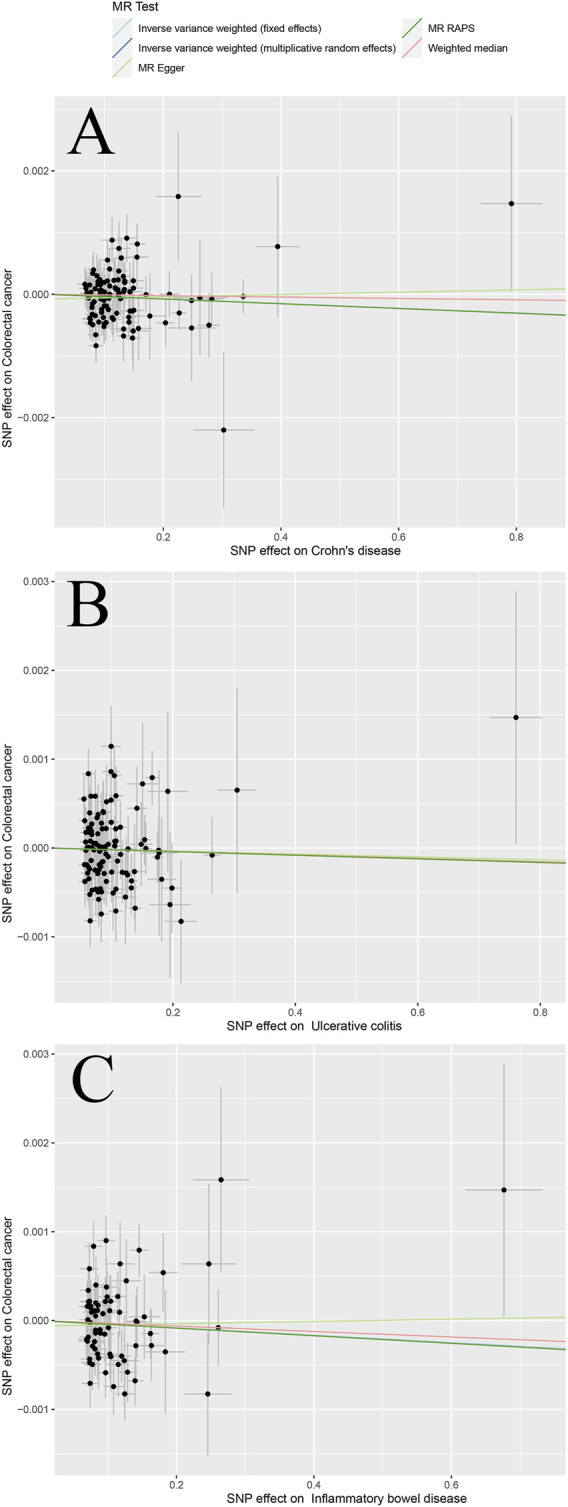
Scatter plots of different MR outcomes. Note: Scatter plots showed the causal effect of exposure on CRC **(A)** Crohn’s disease against colorectal cancer risk **(B)** ulcerative colitis against colorectal cancer risk; and **(C)** inflammatory bowel disease against colorectal cancer risk. The slopes of each line represent the causal association for each method.

**FIGURE 3:**
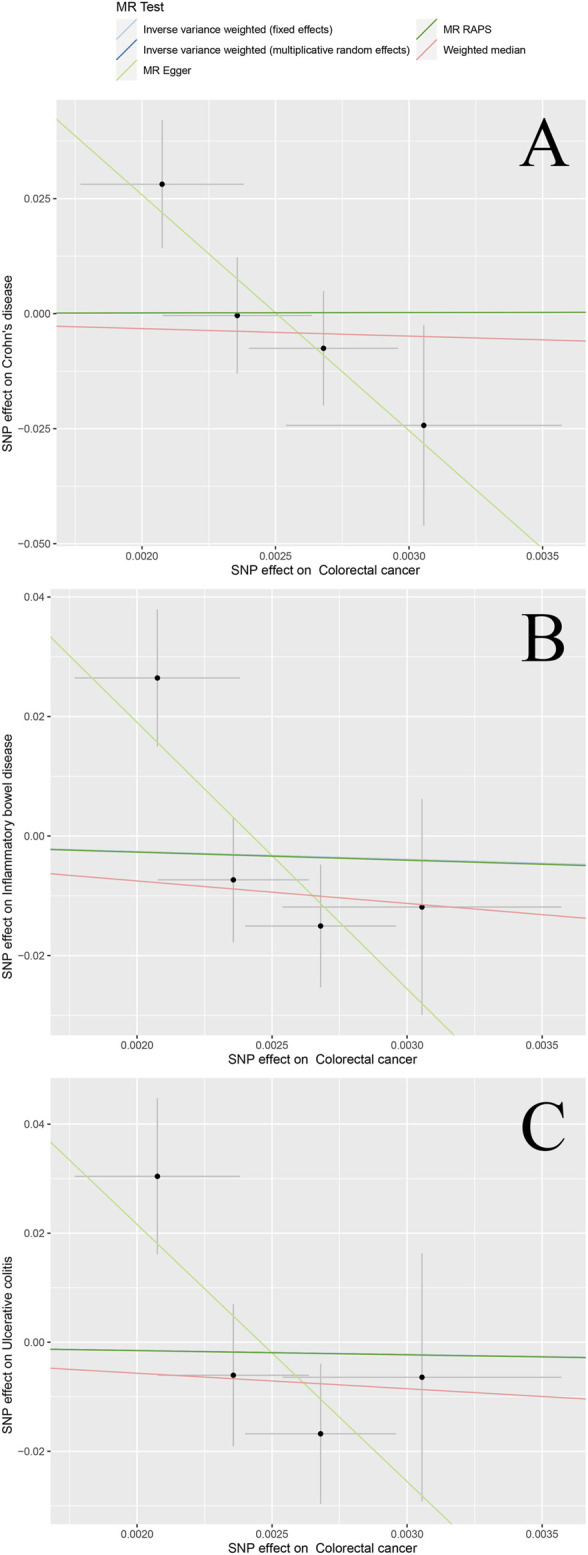
Scatter plots of different bidirectional MR outcomes. Note: Scatter plots showed the causal effect of exposure on CRC **(A)** Colorectal cancer against Crohn’s disease risk **(B)** colorectal cancer against ulcerative colitis risk; and **(C)** colorectal cancer against inflammatory bowel disease risk. The slopes of each line represent the causal association for each method.

The scatter plots in Figure 2 and Figure 3 illustrates the effect of each individual SNP on risk factors and outcomes and showed a curve for causal estimates. The leave-one-out test in Supplementary Figures S1–S6 showed that each SNP was robust in the analysis. Supplementary Figure S7 showed forest plots for the results of the final causal estimation. The funnel plots in Supplementary Figure S8 showed the heterogeneity of estimates for each SNP.

## Discussion

To our knowledge, the current study is the first to explore the causal relationship between IBD and colorectal cancer using large GWAS dataset and MR analysis. Our study found that the incidence of IBD, CD, and UC had no causal relationship with colorectal cancer, and also no reverse causation was found. In each group of MR analysis we applied four different MR methods and all reached the same conclusion, indicating that the conclusions are robust.

Traditional observational studies have reported an association between the incidence of IBD, CD, and UC and colorectal cancer. It has been found that patients with UC had an increased risk of developing CRC (Zhang et al., 2015). A larger cohort study also discovered an increased risk of CRC in people with UC compared to people without UC (Olén et al., 2020). A systematic review has confirmed this view further (Keller et al., 2019). In addition, the application of drugs such as 5-aminosalicylic acid (5-ASA), mercaptopurine, non-steroidal anti-inflammatory drugs, and anti-tumor necrosis factor inhibitors in IBD patients can reduce the incidence of CRC in IBD (Wijnands et al., 2021), which also appears to demonstrate an association between IBD and CRC.

The molecular mechanism of IBD-induced CRC received a significant attention of researchers. Field cancerisation is considered an established mechanism for the development of IBD-related CRC (Yalchin et al., 2021). A systematic review found that certain individual genes were hypermethylated in colitis-related cancers: RUNX3, MINT1, MYOD and p16 exon one and promoter regions of EYA4 and ESR, and DNA methylation patterns differ between IBD-related CRC and Sporadic CRC (sCRC) (Emmett et al., 2017). The mutation of tumor suppressor gene tp53 and the expression of p53 protein were closely related to the development of UC-related CRC, which was also confirmed in the subsequent meta-analysis (Lu et al., 2017; Keller et al., 2019; Lucafò et al., 2021; Wijnands et al., 2021).

It was shown that HMGB1 induces proliferation of tumor cell and expression of PCNA through ERK1/2 pathway to promote the occurrence of IBD-related CRC, while GSDME-mediated pyroptosis can release HMGB1. The latter finding sheds light on the link between pyroptosis and IBD-related CRC development (Tan et al., 2020). Further, the overproduction of 5-HT in the gastrointestinal tract promotes IBD-related CRC progression by enhancing NLRP3 inflammasome activation (Li et al., 2021).

However, most of the previous studies are observational, and the causality remains uncertain. Contrary to previous conclusions, our MR analysis outcomes do not support a causal relationship between IBD and CRC.

Because genetic variants associated with risk factors are randomly assigned at birth, the effects of risk factors on individuals are lifelong. This is one of the advantages of MR analysis. Compared with traditional observational studies, MR analysis can provide more reliable evidence because it is less susceptible to confounding factors and reverse causation.

The difference of our conclusions from the conclusions of previous studies may have several possible explanations. First, observational studies cannot remove the influence of certain confounding factors. In a stratified meta-analysis, it was confirmed that IBD patients with PSC or with a family history of CRC were more likely to develop CRC (Wijnands et al., 2021). Although approximately two-thirds of primary sclerosing cholangitis (PSC) patients suffer fom IBD at the same time (Karlsen et al., 2017), PSC patients also have an increased risk of developing colorectal cancer. It is difficult to say whether it is PSC or IBD that promotes CRC. Having a family history of CRC may also be a confounding factor for the same reason (Wijnands et al., 2021). In this MR study, SNPs related to confounding factors such as PSC were removed through PhenoScanner screening (Staley et al., 2016), and would not be affected by confounding.

**FIGURE 4:**
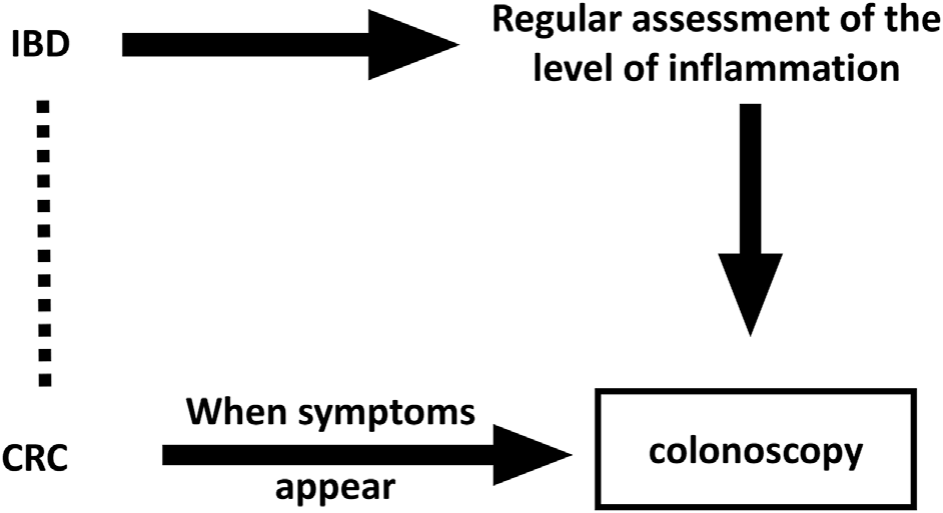
Directed acyclic graph with underlying detection bias in observational studies. The Dashed line indicates wrong associations that might be drawn in observational studies.

Second, a knowledge of exposure history increases disease detection efforts in the exposed, suggesting that observational studies may be biased in the detection of symptoms (Zi-yan et al., 2019). Regular colonoscopy detection work in IBD patients has become the norm, which will increase the detection rate of CRC. This, in turn, makes the conclusion implausible, similar to the conclusion on estrogen treatment in endometrial cancer (Horwitz and Feinstein, 1978; Greenland and Neutra, 1981). The detection bias is shown in Figure 4.

Finally, the genetic mechanisms of IBD-related CRC may differ from those of sCRC. In terms of clinicopathological characteristics, compared with sporadic CRC, IBD-related CRC has an increased incidence of multiple tumors, poor differentiation, and a lower incidence of rectal cancer (Reynolds et al., 2017). At the molecular level, it has been found that IBD-associated CRC differs from sporadic CRC in DNA methylation patterns (Emmett et al., 2017). Instead of the typical epithelial tumor subtype associated with WNT signaling, the IBD-related CRC subtype revealed by transcriptomics is a mesenchymal stroma-rich subtype (Rajamäki et al., 2021). The mutation frequencies of somatic adenomatous polyposis *coli* (APC) and Kirsten rat sarcoma virus (KRAS) are lower in epithelial tumor tissue from patients with IBD-related CRC, while tumor protein P53 (TP53) mutations and Myc proto-oncogene protein (MYC) amplifications are detected earlier during tumor progression in comparison to sCRC (Lucafò et al., 2021; Rajamäki et al., 2021). IBD-related CRC has a unique genetic makeup. Several IBD-related CRC specific genetic mutations have been identified, including mutations in SOX9, EP300, NRG1, and IL16 (Yalchin et al., 2021). The burden of single nucleotide alterations (SNAs) was slightly increased in IBD-related CRC compared to sCRC, where SNAs included recurrent mutations in genes that are not frequently mutated in sCRC (Yalchin et al., 2021). Future research should focus on stratified studies on CRC datasets to explore the association of IBD with specific CRC types.

Another advantage of the current study is the large sample size, which can reduce sampling errors.

Despite advantages, this study has a number of limitations. Since we used the summary level data, the effect of covariates on the results could not be removed by a stratified study. Considering that some types of CRC may be associated with IBD, future studies need to perform stratified analyses based on individual-level data to further clarify the causal relationship between IBD and CRC.

Although epidemiological survey studies have found that advanced age and male gender are risk factors for CRC (Dekker et al., 2019; Keller et al., 2019; Wijnands et al., 2021), the effect of gender and age on this causal relationship was not explored in our study due to data limitations.

The exposure and outcome data in ourstudy were all derived from the European population. Although the impact of population stratification on MR studies was reduced, the causal relationship conclusions drawn from the European population may not necessarily be applicable to other populations, such as Asians.

Some conclusions of this study have low power and may increase the incidence of statistical type 2 error. This probably due to the relatively small sample size of the CRC. Further, since the ratio estimation method assumes a linear relationship between exposure and outcome, this study cannot rule out the possibility of a non-linear relationship between the two. Finally, MR research inevitably has individual canalization. For example, because individuals have a high risk of inflammatory bowel disease, the body compensates for the inflammatory response through various channels.

Still, it is worth noting that as long as the SNPs used in this study satisfy the three assumptions of the instrumental variables, the MR conclusions obtained are still valid.

## Conclusion

Using a two-sample bidirectional MR analysis, we did not find a causal or reverse causal relationship between the incidence of IBD and the incidence of CRC in populations of European ancestry.

## Data Availability

Publicly available datasets were analyzed in this study. This data can be found here: https://gwas.mrcieu.ac.uk/datasets/ieu-a-12/ https://gwas.mrcieu.ac.uk/datasets/ieu-a-970/ https://gwas.mrcieu.ac.uk/datasets/ieu-a-294/ https://gwas.mrcieu.ac.uk/datasets/ieu-b-4965/.
