# Supplementary material for: A mendelian randomization study with populations of European ancestry rules out a causal relationship between inflammatory bowel disease and colorectal cancer
Source: Front Genet. 2022 Aug 24;13:949325. doi: 10.3389/fgene.2022.949325 (PMC9449310; doi:10.3389/fgene.2022.949325)
Supplement: Supplementary file 1 [file DataSheet1.docx]

**Supplemental Table S1.** **Characteristics of the SNPs used in the Mendelian randomization analysis of the effects of crohn’s disease on colorectal cancer risk**

| SNP | Effects on crohn’s disease | | | | | | Effects on colorectal cancer | | | | | | Chr | Position |
| --- | --- | --- | --- | --- | --- | --- | --- | --- | --- | --- | --- | --- | --- | --- |
|  | EA | OA | EAF | Beta | SE | p-val | EA | OA | EAF | Beta | SE | p-val |  |  |
| rs10798069 | T | G | 0.4925 | -0.0704 | 0.0120 | 4.25E-09 | T | G | 0.4839 | 0.0000 | 0.0003 | 0.9400 | 1 | 186875459 |
| rs10800309 | G | A | 0.6578 | -0.0904 | 0.0126 | 8.48E-13 | G | A | 0.6820 | 0.0004 | 0.0003 | 0.1300 | 1 | 161472158 |
| rs12041056 | T | C | 0.4118 | 0.1084 | 0.0121 | 3.51E-19 | T | C | 0.4164 | 0.0000 | 0.0003 | 1.0000 | 1 | 67627260 |
| rs12411259 | A | G | 0.2401 | 0.1344 | 0.0137 | 1.43E-22 | A | G | 0.2423 | 0.0002 | 0.0003 | 0.5600 | 1 | 172866210 |
| rs2488401 | T | C | 0.2040 | 0.1205 | 0.0147 | 2.07E-16 | T | C | 0.2078 | 0.0001 | 0.0003 | 0.8200 | 1 | 197781198 |
| rs36016881 | G | A | 0.1754 | -0.1090 | 0.0170 | 1.60E-10 | G | A | 0.1746 | -0.0001 | 0.0004 | 0.7100 | 1 | 8051241 |
| rs4655709 | A | G | 0.7377 | 0.1208 | 0.0138 | 2.40E-18 | A | G | 0.7389 | -0.0003 | 0.0003 | 0.3400 | 1 | 67903316 |
| rs7517847 | G | T | 0.4352 | -0.3358 | 0.0125 | 1.38E-159 | G | T | 0.4435 | 0.0000 | 0.0003 | 0.9000 | 1 | 67681669 |
| rs76181804 | G | A | 0.1065 | 0.1245 | 0.0193 | 1.13E-10 | G | A | 0.1177 | 0.0007 | 0.0004 | 0.0870 | 1 | 198601705 |
| rs11679753 | G | A | 0.6168 | -0.0825 | 0.0125 | 4.57E-11 | G | A | 0.6242 | 0.0001 | 0.0003 | 0.6700 | 2 | 62554466 |
| rs11691685 | G | A | 0.0802 | -0.1577 | 0.0233 | 1.35E-11 | G | A | 0.0765 | 0.0006 | 0.0005 | 0.3000 | 2 | 145481827 |
| rs13001325 | T | C | 0.3757 | -0.1227 | 0.0126 | 1.68E-22 | T | C | 0.3874 | -0.0001 | 0.0003 | 0.8000 | 2 | 102939036 |
| rs1517352 | C | A | 0.6048 | 0.0800 | 0.0125 | 1.31E-10 | C | A | 0.6259 | -0.0005 | 0.0003 | 0.0890 | 2 | 191931464 |
| rs35320439 | C | T | 0.3104 | 0.0841 | 0.0138 | 9.89E-10 | C | T | 0.3118 | 0.0001 | 0.0003 | 0.7600 | 2 | 242737341 |
| rs6740462 | A | C | 0.7378 | 0.0997 | 0.0141 | 1.74E-12 | A | C | 0.7345 | -0.0002 | 0.0003 | 0.5500 | 2 | 65667272 |
| rs925255 | T | C | 0.4517 | -0.1000 | 0.0121 | 1.07E-16 | T | C | 0.4780 | 0.0000 | 0.0003 | 0.9000 | 2 | 28614794 |
| rs11713774 | C | T | 0.1427 | 0.1327 | 0.0172 | 1.09E-14 | C | T | 0.1543 | 0.0000 | 0.0004 | 0.9100 | 3 | 18765978 |
| rs10055349 | A | G | 0.2189 | 0.1557 | 0.0142 | 4.72E-28 | A | G | 0.2328 | 0.0008 | 0.0003 | 0.0140 | 5 | 40441718 |
| rs1363907 | A | G | 0.4212 | 0.1026 | 0.0126 | 3.89E-16 | A | G | 0.4350 | -0.0005 | 0.0003 | 0.1000 | 5 | 96252803 |
| rs1567009 | A | G | 0.5834 | 0.1050 | 0.0122 | 9.24E-18 | A | G | 0.5868 | 0.0002 | 0.0003 | 0.4500 | 5 | 40002579 |
| rs16870166 | A | G | 0.0504 | 0.1488 | 0.0262 | 1.36E-08 | A | G | 0.0482 | -0.0006 | 0.0007 | 0.3600 | 5 | 40607868 |
| rs181826 | A | C | 0.6266 | 0.0996 | 0.0127 | 4.53E-15 | A | C | 0.6181 | -0.0005 | 0.0003 | 0.0970 | 5 | 141526057 |
| rs1896707 | T | C | 0.0826 | 0.2776 | 0.0197 | 3.29E-45 | T | C | 0.0780 | -0.0005 | 0.0005 | 0.3400 | 5 | 150245129 |
| rs34804116 | A | C | 0.3867 | -0.0939 | 0.0127 | 1.27E-13 | A | C | 0.3816 | -0.0001 | 0.0003 | 0.8100 | 5 | 72539850 |
| rs3776414 | G | T | 0.3756 | 0.0888 | 0.0123 | 5.04E-13 | G | T | 0.3542 | 0.0002 | 0.0003 | 0.5800 | 5 | 10752315 |
| rs395157 | T | C | 0.4842 | 0.0981 | 0.0120 | 2.36E-16 | T | C | 0.4901 | -0.0004 | 0.0003 | 0.1800 | 5 | 38867732 |
| rs4703855 | T | C | 0.2998 | -0.0733 | 0.0132 | 3.03E-08 | T | C | 0.2953 | 0.0000 | 0.0003 | 0.9400 | 5 | 71693899 |
| rs56163845 | G | A | 0.3096 | -0.0919 | 0.0135 | 9.40E-12 | G | A | 0.3021 | -0.0002 | 0.0003 | 0.4700 | 5 | 173373948 |
| rs56167332 | A | C | 0.3375 | 0.1706 | 0.0127 | 2.29E-41 | A | C | 0.3450 | 0.0000 | 0.0003 | 0.9900 | 5 | 158827769 |
| rs71624119 | A | G | 0.2422 | -0.0923 | 0.0149 | 6.57E-10 | A | G | 0.2403 | 0.0000 | 0.0003 | 0.9700 | 5 | 55440730 |
| rs7711427 | C | A | 0.6130 | 0.2480 | 0.0125 | 5.17E-88 | C | A | 0.6046 | -0.0001 | 0.0003 | 0.7200 | 5 | 40414886 |
| rs11152949 | G | A | 0.3195 | 0.1338 | 0.0129 | 2.18E-25 | G | A | 0.3266 | 0.0001 | 0.0003 | 0.8000 | 6 | 106449085 |
| rs116675765 | T | C | 0.0318 | -0.2481 | 0.0403 | 7.75E-10 | T | C | 0.0269 | 0.0005 | 0.0009 | 0.5300 | 6 | 30057544 |
| rs12194548 | G | A | 0.1611 | -0.0929 | 0.0170 | 4.61E-08 | G | A | 0.1519 | -0.0002 | 0.0004 | 0.7000 | 6 | 128056974 |
| rs1267501 | C | T | 0.8106 | 0.0872 | 0.0152 | 9.69E-09 | C | T | 0.8063 | 0.0003 | 0.0004 | 0.4000 | 6 | 14715257 |
| rs13204048 | C | T | 0.3880 | -0.0685 | 0.0123 | 2.89E-08 | C | T | 0.3899 | -0.0001 | 0.0003 | 0.7400 | 6 | 3420406 |
| rs3130186 | T | C | 0.2396 | 0.0852 | 0.0144 | 3.19E-09 | T | C | 0.2462 | -0.0007 | 0.0003 | 0.0430 | 6 | 33056207 |
| rs367254 | A | C | 0.6217 | -0.0793 | 0.0128 | 5.79E-10 | A | C | 0.6249 | 0.0003 | 0.0003 | 0.2600 | 6 | 111916673 |
| rs6456426 | A | C | 0.4984 | -0.0992 | 0.0120 | 1.37E-16 | A | C | 0.4979 | 0.0005 | 0.0003 | 0.0630 | 6 | 21438889 |
| rs7758080 | G | A | 0.2694 | 0.0761 | 0.0132 | 7.27E-09 | G | A | 0.2691 | -0.0004 | 0.0003 | 0.2000 | 6 | 149577079 |
| rs7773324 | A | G | 0.6002 | 0.0787 | 0.0129 | 1.06E-09 | A | G | 0.6474 | -0.0002 | 0.0003 | 0.4700 | 6 | 382559 |
| rs9457247 | T | C | 0.5398 | 0.1237 | 0.0124 | 2.08E-23 | T | C | 0.5280 | 0.0000 | 0.0003 | 0.9800 | 6 | 167392174 |
| rs9491892 | G | T | 0.1496 | 0.1379 | 0.0164 | 3.80E-17 | G | T | 0.1540 | 0.0009 | 0.0004 | 0.0190 | 6 | 128280358 |
| rs9494844 | A | C | 0.2519 | -0.0889 | 0.0142 | 4.18E-10 | A | C | 0.2348 | -0.0002 | 0.0003 | 0.5300 | 6 | 137983469 |
| rs12718244 | A | G | 0.4081 | 0.0801 | 0.0122 | 4.62E-11 | A | G | 0.4245 | 0.0003 | 0.0003 | 0.2300 | 7 | 50175654 |
| rs2395022 | C | A | 0.9589 | -0.1772 | 0.0282 | 3.13E-10 | C | A | 0.9582 | 0.0004 | 0.0007 | 0.6100 | 7 | 98750379 |
| rs2538470 | G | A | 0.6378 | -0.0750 | 0.0123 | 1.05E-09 | G | A | 0.6193 | -0.0002 | 0.0003 | 0.6000 | 7 | 148220448 |
| rs3801810 | A | G | 0.2337 | 0.1051 | 0.0140 | 6.63E-14 | A | G | 0.2447 | 0.0006 | 0.0003 | 0.0890 | 7 | 26892531 |
| rs4917129 | C | T | 0.5957 | 0.0953 | 0.0125 | 2.08E-14 | C | T | 0.5779 | -0.0002 | 0.0003 | 0.3900 | 7 | 50323174 |
| rs76546301 | A | G | 0.0182 | 0.2253 | 0.0393 | 9.79E-09 | A | G | 0.0182 | 0.0016 | 0.0010 | 0.1300 | 7 | 50498389 |
| rs6651252 | C | T | 0.1300 | -0.1490 | 0.0183 | 3.86E-16 | C | T | 0.1308 | -0.0002 | 0.0004 | 0.6000 | 8 | 129567181 |
| rs7015630 | C | T | 0.2657 | -0.0842 | 0.0138 | 9.00E-10 | C | T | 0.2662 | 0.0003 | 0.0003 | 0.2800 | 8 | 90875918 |
| rs3812609 | T | C | 0.1707 | -0.0938 | 0.0169 | 2.95E-08 | T | C | 0.1669 | 0.0001 | 0.0004 | 0.8500 | 9 | 139408892 |
| rs76606004 | T | C | 0.0108 | 0.3027 | 0.0520 | 5.79E-09 | T | C | 0.0130 | -0.0022 | 0.0013 | 0.0810 | 9 | 117650509 |
| rs7848647 | C | T | 0.6746 | 0.1413 | 0.0130 | 1.55E-27 | C | T | 0.6717 | -0.0004 | 0.0003 | 0.2100 | 9 | 117569046 |
| rs1250573 | A | G | 0.3155 | -0.1417 | 0.0134 | 5.86E-26 | A | G | 0.3302 | 0.0005 | 0.0003 | 0.1300 | 10 | 81042475 |
| rs16917597 | A | G | 0.0209 | -0.2622 | 0.0460 | 1.21E-08 | A | G | 0.0228 | 0.0001 | 0.0009 | 0.9500 | 10 | 64422997 |
| rs2153283 | A | C | 0.2170 | -0.1088 | 0.0155 | 2.39E-12 | A | C | 0.2112 | -0.0004 | 0.0003 | 0.2300 | 10 | 59972299 |
| rs2227551 | T | G | 0.7288 | 0.0994 | 0.0137 | 4.72E-13 | T | G | 0.7195 | 0.0002 | 0.0003 | 0.5400 | 10 | 75669190 |
| rs224140 | T | C | 0.0741 | -0.1325 | 0.0237 | 2.20E-08 | T | C | 0.0733 | 0.0006 | 0.0005 | 0.3000 | 10 | 64473861 |
| rs34779708 | G | T | 0.3512 | 0.1345 | 0.0124 | 1.90E-27 | G | T | 0.3451 | 0.0002 | 0.0003 | 0.4600 | 10 | 35467671 |
| rs41295117 | C | T | 0.1666 | -0.1129 | 0.0174 | 8.90E-11 | C | T | 0.1665 | -0.0009 | 0.0004 | 0.0200 | 10 | 6125322 |
| rs7097656 | C | T | 0.7965 | 0.1321 | 0.0152 | 3.89E-18 | C | T | 0.7952 | 0.0003 | 0.0003 | 0.4000 | 10 | 82250831 |
| rs12796489 | A | C | 0.0229 | -0.7918 | 0.0527 | 4.96E-51 | A | C | 0.0097 | -0.0015 | 0.0014 | 0.3000 | 11 | 3059360 |
| rs34787213 | T | C | 0.1401 | -0.1497 | 0.0183 | 2.85E-16 | T | C | 0.1401 | 0.0003 | 0.0004 | 0.5200 | 11 | 60799046 |
| rs559928 | C | T | 0.8128 | 0.0991 | 0.0158 | 3.75E-10 | C | T | 0.8097 | -0.0001 | 0.0004 | 0.7800 | 11 | 64150370 |
| rs28999107 | T | G | 0.4387 | 0.0857 | 0.0127 | 1.29E-11 | T | G | 0.4507 | -0.0008 | 0.0003 | 0.0034 | 12 | 6493100 |
| rs4767956 | G | A | 0.3875 | -0.0769 | 0.0123 | 4.29E-10 | G | A | 0.3762 | 0.0000 | 0.0003 | 0.9100 | 12 | 40324674 |
| rs76906269 | G | A | 0.0188 | 0.3943 | 0.0370 | 1.75E-26 | G | A | 0.0150 | 0.0008 | 0.0011 | 0.5000 | 12 | 40607709 |
| rs7969592 | G | A | 0.4746 | -0.0732 | 0.0120 | 1.04E-09 | G | A | 0.4725 | 0.0001 | 0.0003 | 0.8300 | 12 | 68579649 |
| rs61959448 | A | G | 0.1506 | 0.1073 | 0.0167 | 1.50E-10 | A | G | 0.1485 | -0.0001 | 0.0004 | 0.8700 | 13 | 42910319 |
| rs6561151 | A | G | 0.2235 | 0.1471 | 0.0142 | 4.68E-25 | A | G | 0.2074 | -0.0003 | 0.0003 | 0.4300 | 13 | 44484706 |
| rs915286 | A | G | 0.5488 | 0.0666 | 0.0120 | 2.59E-08 | A | G | 0.5615 | 0.0002 | 0.0003 | 0.5600 | 13 | 40695992 |
| rs9554587 | G | A | 0.2242 | -0.0952 | 0.0147 | 8.29E-11 | G | A | 0.2122 | 0.0003 | 0.0003 | 0.3900 | 13 | 100040654 |
| rs11159833 | T | C | 0.0868 | 0.1552 | 0.0208 | 7.59E-14 | T | C | 0.0914 | 0.0006 | 0.0005 | 0.2100 | 14 | 88476004 |
| rs1569328 | T | C | 0.1702 | -0.1092 | 0.0167 | 6.47E-11 | T | C | 0.1719 | 0.0000 | 0.0004 | 0.9900 | 14 | 75741751 |
| rs17293632 | T | C | 0.2364 | 0.1284 | 0.0140 | 3.70E-20 | T | C | 0.2358 | 0.0006 | 0.0003 | 0.0740 | 15 | 67442596 |
| rs11117431 | G | A | 0.1978 | -0.1493 | 0.0164 | 1.09E-19 | G | A | 0.1955 | -0.0001 | 0.0004 | 0.7800 | 16 | 86015316 |
| rs1486418 | A | G | 0.2773 | 0.0750 | 0.0134 | 2.22E-08 | A | G | 0.2782 | -0.0004 | 0.0003 | 0.2100 | 16 | 51043549 |
| rs1528602 | C | T | 0.2620 | -0.1161 | 0.0136 | 1.72E-17 | C | T | 0.2616 | -0.0002 | 0.0003 | 0.4800 | 16 | 50999201 |
| rs1990623 | G | A | 0.1543 | 0.2037 | 0.0158 | 4.52E-38 | G | A | 0.1501 | -0.0005 | 0.0004 | 0.2400 | 16 | 50565970 |
| rs1990684 | T | C | 0.1095 | -0.1182 | 0.0198 | 2.47E-09 | T | C | 0.1049 | -0.0001 | 0.0005 | 0.8300 | 16 | 50427554 |
| rs2058813 | C | T | 0.8097 | -0.1151 | 0.0148 | 6.42E-15 | C | T | 0.8054 | 0.0004 | 0.0004 | 0.2500 | 16 | 50129454 |
| rs2270395 | T | C | 0.7616 | 0.1243 | 0.0145 | 8.93E-18 | T | C | 0.7727 | 0.0004 | 0.0003 | 0.2700 | 16 | 50846832 |
| rs4643314 | T | C | 0.4978 | 0.1247 | 0.0119 | 9.25E-26 | T | C | 0.4879 | 0.0000 | 0.0003 | 0.9600 | 16 | 50375955 |
| rs7194886 | T | C | 0.4357 | -0.2270 | 0.0122 | 1.42E-77 | T | C | 0.4185 | 0.0003 | 0.0003 | 0.2800 | 16 | 50725193 |
| rs7195296 | C | A | 0.2692 | -0.0921 | 0.0138 | 2.56E-11 | C | A | 0.2663 | -0.0001 | 0.0003 | 0.8500 | 16 | 11749774 |
| rs2945412 | A | G | 0.5866 | 0.1130 | 0.0123 | 4.51E-20 | A | G | 0.5860 | -0.0004 | 0.0003 | 0.1800 | 17 | 25843643 |
| rs3853824 | C | T | 0.6385 | 0.0814 | 0.0126 | 1.17E-10 | C | T | 0.6580 | 0.0004 | 0.0003 | 0.1800 | 17 | 54880993 |
| rs4795397 | G | A | 0.4713 | 0.1323 | 0.0120 | 3.84E-28 | G | A | 0.4737 | -0.0007 | 0.0003 | 0.0160 | 17 | 38026035 |
| rs9889296 | A | G | 0.2723 | -0.1431 | 0.0138 | 2.96E-25 | A | G | 0.2742 | 0.0000 | 0.0003 | 0.9000 | 17 | 32583911 |
| rs7236492 | T | C | 0.1537 | -0.0997 | 0.0173 | 9.09E-09 | T | C | 0.1719 | 0.0000 | 0.0004 | 0.9200 | 18 | 77220616 |
| rs12720356 | C | A | 0.0857 | 0.1433 | 0.0216 | 3.39E-11 | C | A | 0.0969 | 0.0000 | 0.0005 | 0.9300 | 19 | 10469975 |
| rs17694108 | A | G | 0.2797 | 0.0796 | 0.0135 | 3.29E-09 | A | G | 0.2760 | -0.0002 | 0.0003 | 0.6300 | 19 | 33731551 |
| rs2024092 | A | G | 0.2162 | 0.1477 | 0.0143 | 7.13E-25 | A | G | 0.2009 | -0.0007 | 0.0003 | 0.0420 | 19 | 1124031 |
| rs35164067 | A | G | 0.2039 | -0.1434 | 0.0156 | 3.19E-20 | A | G | 0.2018 | 0.0003 | 0.0003 | 0.4300 | 19 | 10525181 |
| rs640466 | C | T | 0.3739 | -0.0758 | 0.0125 | 1.31E-09 | C | T | 0.3697 | 0.0005 | 0.0003 | 0.1100 | 19 | 34670725 |
| rs752508 | A | G | 0.2833 | 0.1066 | 0.0137 | 7.95E-15 | A | G | 0.2924 | -0.0002 | 0.0003 | 0.4200 | 19 | 1168753 |
| rs259964 | G | A | 0.5414 | -0.0713 | 0.0119 | 2.08E-09 | G | A | 0.5454 | 0.0000 | 0.0003 | 0.9900 | 20 | 57824309 |
| rs6074022 | T | C | 0.7497 | -0.0963 | 0.0138 | 2.70E-12 | T | C | 0.7546 | -0.0001 | 0.0003 | 0.8700 | 20 | 44740196 |
| rs6111031 | T | C | 0.1591 | -0.2824 | 0.0181 | 9.61E-55 | T | C | 0.1215 | 0.0001 | 0.0004 | 0.8500 | 20 | 1682037 |
| rs1297258 | T | C | 0.4249 | -0.1272 | 0.0122 | 2.11E-25 | T | C | 0.4304 | 0.0001 | 0.0003 | 0.8000 | 21 | 16806709 |
| rs2284553 | G | A | 0.5904 | 0.1032 | 0.0123 | 5.63E-17 | G | A | 0.5897 | -0.0002 | 0.0003 | 0.5100 | 21 | 34776695 |
| rs8127691 | C | T | 0.6132 | -0.1234 | 0.0122 | 4.48E-24 | C | T | 0.6111 | -0.0002 | 0.0003 | 0.4100 | 21 | 45614860 |
| rs2413583 | T | C | 0.1654 | -0.2104 | 0.0168 | 7.72E-36 | T | C | 0.1646 | 0.0000 | 0.0004 | 1.0000 | 22 | 39659773 |
| rs727563 | T | C | 0.7973 | -0.0921 | 0.0145 | 1.88E-10 | T | C | 0.8039 | -0.0002 | 0.0004 | 0.5000 | 22 | 41902503 |

Abbreviation: EA, Effect Allele; OA, Other Allele; EAF, effect allele frequency; SE, standard error; SNP, single nucleotide polymorphism; Chr, Chromosome

**Supplemental Table S2.** **Characteristics of the SNPs used in the Mendelian randomization analysis of the effects of ulcerative colitis on colorectal cancer risk**

| SNP | Effects on ulcerative colitis | | | | | | Effects on colorectal cancer | | | | | | Chr | Position |
| --- | --- | --- | --- | --- | --- | --- | --- | --- | --- | --- | --- | --- | --- | --- |
|  | EA | OA | EAF | Beta | SE | p-val | EA | OA | EAF | Beta | SE | p-val |  |  |
| rs10800309 | G | A | 0.6578 | -0.1321 | 0.0104 | 6.15E-37 | G | A | 0.6820 | 0.0004 | 0.0003 | 0.1300 | 1 | 161472158 |
| rs12103 | C | T | 0.8166 | -0.0867 | 0.0131 | 3.28E-11 | C | T | 0.8049 | -0.0004 | 0.0004 | 0.2600 | 1 | 1247494 |
| rs12411259 | A | G | 0.2401 | 0.0669 | 0.0115 | 6.18E-09 | A | G | 0.2423 | 0.0002 | 0.0003 | 0.5600 | 1 | 172866210 |
| rs17668708 | T | C | 0.0996 | 0.0992 | 0.0163 | 1.29E-09 | T | C | 0.1060 | 0.0011 | 0.0005 | 0.0120 | 1 | 198640488 |
| rs2297559 | A | G | 0.6822 | 0.0742 | 0.0110 | 1.88E-11 | A | G | 0.6875 | -0.0005 | 0.0003 | 0.1200 | 1 | 160854526 |
| rs2488401 | T | C | 0.2040 | 0.1071 | 0.0122 | 1.43E-18 | T | C | 0.2078 | 0.0001 | 0.0003 | 0.8200 | 1 | 197781198 |
| rs2769267 | A | G | 0.7509 | 0.0680 | 0.0116 | 4.10E-09 | A | G | 0.7551 | -0.0005 | 0.0003 | 0.1400 | 1 | 151333167 |
| rs2816958 | G | A | 0.8852 | 0.0990 | 0.0158 | 3.64E-10 | G | A | 0.8868 | 0.0005 | 0.0004 | 0.2200 | 1 | 200101920 |
| rs34856868 | A | G | 0.0316 | -0.1953 | 0.0341 | 9.80E-09 | A | G | 0.0297 | 0.0006 | 0.0008 | 0.4400 | 1 | 92554283 |
| rs4845140 | T | C | 0.0412 | -0.2134 | 0.0262 | 4.33E-16 | T | C | 0.0428 | 0.0008 | 0.0007 | 0.2300 | 1 | 206970470 |
| rs4845604 | A | G | 0.1483 | -0.1236 | 0.0145 | 1.21E-17 | A | G | 0.1462 | 0.0003 | 0.0004 | 0.4800 | 1 | 151801680 |
| rs55693740 | A | G | 0.0662 | 0.1373 | 0.0204 | 1.54E-11 | A | G | 0.0709 | -0.0003 | 0.0005 | 0.6300 | 1 | 67770278 |
| rs6025 | C | T | 0.9706 | 0.1775 | 0.0319 | 2.51E-08 | C | T | 0.9769 | -0.0001 | 0.0009 | 0.9500 | 1 | 169519049 |
| rs6588248 | G | T | 0.5297 | 0.0820 | 0.0099 | 1.38E-16 | G | T | 0.5311 | 0.0000 | 0.0003 | 0.9600 | 1 | 67652984 |
| rs72634258 | C | T | 0.1757 | -0.1269 | 0.0140 | 1.25E-19 | C | T | 0.1743 | 0.0000 | 0.0004 | 0.9800 | 1 | 8150638 |
| rs11691685 | G | A | 0.0802 | -0.1225 | 0.0188 | 7.27E-11 | G | A | 0.0765 | 0.0006 | 0.0005 | 0.3000 | 2 | 145481827 |
| rs1517352 | C | A | 0.6048 | 0.0779 | 0.0103 | 3.87E-14 | C | A | 0.6259 | -0.0005 | 0.0003 | 0.0890 | 2 | 191931464 |
| rs35256947 | C | T | 0.2594 | 0.0822 | 0.0113 | 3.87E-13 | C | T | 0.2499 | -0.0002 | 0.0003 | 0.5200 | 2 | 231171423 |
| rs4664304 | G | A | 0.5621 | -0.0560 | 0.0101 | 2.61E-08 | G | A | 0.5530 | -0.0003 | 0.0003 | 0.2700 | 2 | 160794008 |
| rs4973341 | T | C | 0.6627 | 0.0662 | 0.0104 | 2.03E-10 | T | C | 0.6641 | 0.0006 | 0.0003 | 0.0490 | 2 | 228660362 |
| rs6740462 | A | C | 0.7378 | 0.0800 | 0.0116 | 5.59E-12 | A | C | 0.7345 | -0.0002 | 0.0003 | 0.5500 | 2 | 65667272 |
| rs72924296 | G | A | 0.2696 | -0.0638 | 0.0113 | 1.44E-08 | G | A | 0.2814 | -0.0002 | 0.0003 | 0.5700 | 2 | 199543967 |
| rs925255 | T | C | 0.4517 | -0.0759 | 0.0100 | 2.70E-14 | T | C | 0.4780 | 0.0000 | 0.0003 | 0.9000 | 2 | 28614794 |
| rs11713774 | C | T | 0.1427 | 0.0943 | 0.0143 | 3.92E-11 | C | T | 0.1543 | 0.0000 | 0.0004 | 0.9100 | 3 | 18765978 |
| rs13107612 | T | C | 0.2969 | 0.0733 | 0.0109 | 1.62E-11 | T | C | 0.3116 | 0.0006 | 0.0003 | 0.0540 | 4 | 102739980 |
| rs4692386 | C | T | 0.5930 | 0.0580 | 0.0102 | 1.21E-08 | C | T | 0.5926 | 0.0002 | 0.0003 | 0.5400 | 4 | 26132361 |
| rs10055349 | A | G | 0.2189 | 0.1044 | 0.0118 | 1.13E-18 | A | G | 0.2328 | 0.0008 | 0.0003 | 0.0140 | 5 | 40441718 |
| rs1363907 | A | G | 0.4212 | 0.0815 | 0.0104 | 4.87E-15 | A | G | 0.4350 | -0.0005 | 0.0003 | 0.1000 | 5 | 96252803 |
| rs1567009 | A | G | 0.5834 | 0.0846 | 0.0100 | 3.90E-17 | A | G | 0.5868 | 0.0002 | 0.0003 | 0.4500 | 5 | 40002579 |
| rs17800987 | G | A | 0.0855 | 0.1985 | 0.0170 | 1.67E-31 | G | A | 0.0819 | -0.0005 | 0.0005 | 0.3800 | 5 | 150323428 |
| rs181826 | A | C | 0.6266 | 0.0820 | 0.0104 | 4.05E-15 | A | C | 0.6181 | -0.0005 | 0.0003 | 0.0970 | 5 | 141526057 |
| rs254562 | G | A | 0.4051 | 0.0618 | 0.0100 | 6.69E-10 | G | A | 0.4093 | 0.0008 | 0.0003 | 0.0033 | 5 | 134441457 |
| rs272882 | T | G | 0.6733 | 0.1661 | 0.0109 | 1.47E-52 | T | G | 0.7062 | 0.0008 | 0.0003 | 0.0098 | 5 | 131669161 |
| rs34804116 | A | C | 0.3867 | -0.0575 | 0.0104 | 3.62E-08 | A | C | 0.3816 | -0.0001 | 0.0003 | 0.8100 | 5 | 72539850 |
| rs3776414 | G | T | 0.3756 | 0.0774 | 0.0102 | 2.65E-14 | G | T | 0.3542 | 0.0002 | 0.0003 | 0.5800 | 5 | 10752315 |
| rs395157 | T | C | 0.4842 | 0.0914 | 0.0099 | 2.22E-20 | T | C | 0.4901 | -0.0004 | 0.0003 | 0.1800 | 5 | 38867732 |
| rs4703855 | T | C | 0.2998 | -0.0711 | 0.0109 | 7.16E-11 | T | C | 0.2953 | 0.0000 | 0.0003 | 0.9400 | 5 | 71693899 |
| rs56163845 | G | A | 0.3096 | -0.0649 | 0.0111 | 4.71E-09 | G | A | 0.3021 | -0.0002 | 0.0003 | 0.4700 | 5 | 173373948 |
| rs56167332 | A | C | 0.3375 | 0.1559 | 0.0105 | 7.17E-50 | A | C | 0.3450 | 0.0000 | 0.0003 | 0.9900 | 5 | 158827769 |
| rs7711427 | C | A | 0.6130 | 0.1748 | 0.0102 | 4.63E-66 | C | A | 0.6046 | -0.0001 | 0.0003 | 0.7200 | 5 | 40414886 |
| rs11152949 | G | A | 0.3195 | 0.1051 | 0.0107 | 7.25E-23 | G | A | 0.3266 | 0.0001 | 0.0003 | 0.8000 | 6 | 106449085 |
| rs13204742 | T | G | 0.1267 | 0.0916 | 0.0148 | 5.39E-10 | T | G | 0.1312 | 0.0005 | 0.0004 | 0.2100 | 6 | 128245765 |
| rs17119 | A | G | 0.8060 | 0.0833 | 0.0124 | 2.18E-11 | A | G | 0.8027 | 0.0003 | 0.0004 | 0.4300 | 6 | 14719496 |
| rs6456426 | A | C | 0.4984 | -0.0643 | 0.0099 | 8.18E-11 | A | C | 0.4979 | 0.0005 | 0.0003 | 0.0630 | 6 | 21438889 |
| rs72978783 | T | C | 0.0480 | -0.1504 | 0.0255 | 3.68E-09 | T | C | 0.0435 | -0.0007 | 0.0007 | 0.3000 | 6 | 138094303 |
| rs7773324 | A | G | 0.6002 | 0.0618 | 0.0106 | 5.84E-09 | A | G | 0.6474 | -0.0002 | 0.0003 | 0.4700 | 6 | 382559 |
| rs9457247 | T | C | 0.5398 | 0.0892 | 0.0102 | 2.48E-18 | T | C | 0.5280 | 0.0000 | 0.0003 | 0.9800 | 6 | 167392174 |
| rs12718244 | A | G | 0.4081 | 0.0762 | 0.0100 | 3.35E-14 | A | G | 0.4245 | 0.0003 | 0.0003 | 0.2300 | 7 | 50175654 |
| rs2108225 | A | G | 0.4385 | -0.0675 | 0.0100 | 1.27E-11 | A | G | 0.4236 | -0.0003 | 0.0003 | 0.3400 | 7 | 107453103 |
| rs2395022 | C | A | 0.9589 | -0.1816 | 0.0234 | 8.27E-15 | C | A | 0.9582 | 0.0004 | 0.0007 | 0.6100 | 7 | 98750379 |
| rs2538470 | G | A | 0.6378 | -0.0676 | 0.0102 | 3.00E-11 | G | A | 0.6193 | -0.0002 | 0.0003 | 0.6000 | 7 | 148220448 |
| rs3801835 | T | C | 0.3447 | 0.0641 | 0.0106 | 1.47E-09 | T | C | 0.3401 | 0.0002 | 0.0003 | 0.5100 | 7 | 26852053 |
| rs4917129 | C | T | 0.5957 | 0.0794 | 0.0103 | 9.48E-15 | C | T | 0.5779 | -0.0002 | 0.0003 | 0.3900 | 7 | 50323174 |
| rs6651252 | C | T | 0.1300 | -0.0908 | 0.0148 | 9.08E-10 | C | T | 0.1308 | -0.0002 | 0.0004 | 0.6000 | 8 | 129567181 |
| rs7011507 | A | G | 0.1233 | -0.0846 | 0.0151 | 2.03E-08 | A | G | 0.1227 | 0.0002 | 0.0004 | 0.7200 | 8 | 49129242 |
| rs7015630 | C | T | 0.2657 | -0.0628 | 0.0113 | 2.90E-08 | C | T | 0.2662 | 0.0003 | 0.0003 | 0.2800 | 8 | 90875918 |
| rs4743820 | T | C | 0.7019 | 0.0640 | 0.0109 | 3.80E-09 | T | C | 0.7005 | -0.0002 | 0.0003 | 0.4400 | 9 | 93933158 |
| rs7848647 | C | T | 0.6746 | 0.1324 | 0.0107 | 3.16E-35 | C | T | 0.6717 | -0.0004 | 0.0003 | 0.2100 | 9 | 117569046 |
| rs10761659 | G | A | 0.5399 | 0.1538 | 0.0100 | 4.97E-53 | G | A | 0.5389 | 0.0001 | 0.0003 | 0.7400 | 10 | 64445564 |
| rs11190087 | G | A | 0.5851 | -0.0547 | 0.0100 | 4.21E-08 | G | A | 0.5839 | -0.0006 | 0.0003 | 0.0530 | 10 | 101185830 |
| rs12722515 | A | C | 0.1627 | -0.0989 | 0.0143 | 4.57E-12 | A | C | 0.1627 | -0.0009 | 0.0004 | 0.0230 | 10 | 6081230 |
| rs2153283 | A | C | 0.2170 | -0.0860 | 0.0127 | 1.54E-11 | A | C | 0.2112 | -0.0004 | 0.0003 | 0.2300 | 10 | 59972299 |
| rs2274351 | T | C | 0.5373 | 0.0605 | 0.0104 | 6.93E-09 | T | C | 0.5431 | 0.0000 | 0.0003 | 0.9700 | 10 | 104264107 |
| rs2393904 | T | C | 0.3145 | -0.0788 | 0.0109 | 6.01E-13 | T | C | 0.3119 | 0.0006 | 0.0003 | 0.0590 | 10 | 64379326 |
| rs2688608 | T | G | 0.5570 | 0.0624 | 0.0099 | 2.75E-10 | T | G | 0.5508 | 0.0002 | 0.0003 | 0.4400 | 10 | 75658349 |
| rs34779708 | G | T | 0.3512 | 0.1067 | 0.0102 | 2.07E-25 | G | T | 0.3451 | 0.0002 | 0.0003 | 0.4600 | 10 | 35467671 |
| rs7097656 | C | T | 0.7965 | 0.0993 | 0.0124 | 1.27E-15 | C | T | 0.7952 | 0.0003 | 0.0003 | 0.4000 | 10 | 82250831 |
| rs11230563 | T | C | 0.3480 | -0.0812 | 0.0106 | 1.71E-14 | T | C | 0.3419 | 0.0000 | 0.0003 | 0.9400 | 11 | 60776209 |
| rs12796489 | A | C | 0.0229 | -0.7604 | 0.0432 | 2.87E-69 | A | C | 0.0097 | -0.0015 | 0.0014 | 0.3000 | 11 | 3059360 |
| rs559928 | C | T | 0.8128 | 0.0944 | 0.0130 | 3.33E-13 | C | T | 0.8097 | -0.0001 | 0.0004 | 0.7800 | 11 | 64150370 |
| rs648541 | G | A | 0.3409 | -0.0649 | 0.0107 | 1.22E-09 | G | A | 0.3293 | 0.0008 | 0.0003 | 0.0059 | 11 | 114429934 |
| rs12318183 | A | C | 0.3854 | 0.1095 | 0.0101 | 1.67E-27 | A | C | 0.3882 | -0.0001 | 0.0003 | 0.6100 | 12 | 68503836 |
| rs1388585 | A | G | 0.9808 | -0.3049 | 0.0317 | 6.85E-22 | A | G | 0.9851 | -0.0007 | 0.0012 | 0.5700 | 12 | 40531691 |
| rs12585310 | A | G | 0.3136 | 0.0706 | 0.0108 | 5.25E-11 | A | G | 0.3144 | 0.0001 | 0.0003 | 0.8600 | 13 | 27528347 |
| rs61959439 | T | C | 0.1508 | 0.0905 | 0.0139 | 8.15E-11 | T | C | 0.1483 | -0.0001 | 0.0004 | 0.8600 | 13 | 42862423 |
| rs6561151 | A | G | 0.2235 | 0.1000 | 0.0119 | 3.53E-17 | A | G | 0.2074 | -0.0003 | 0.0003 | 0.4300 | 13 | 44484706 |
| rs941823 | C | T | 0.7509 | 0.0830 | 0.0115 | 6.19E-13 | C | T | 0.7456 | -0.0007 | 0.0003 | 0.0210 | 13 | 41013977 |
| rs9557207 | G | A | 0.2231 | -0.0878 | 0.0121 | 3.52E-13 | G | A | 0.2110 | 0.0003 | 0.0003 | 0.4000 | 13 | 100036418 |
| rs10142466 | G | A | 0.5065 | -0.0580 | 0.0101 | 1.08E-08 | G | A | 0.5049 | 0.0000 | 0.0003 | 0.9200 | 14 | 69271784 |
| rs1569328 | T | C | 0.1702 | -0.0810 | 0.0137 | 3.21E-09 | T | C | 0.1719 | 0.0000 | 0.0004 | 0.9900 | 14 | 75741751 |
| rs55808324 | A | G | 0.0932 | 0.1412 | 0.0168 | 5.13E-17 | A | G | 0.0986 | 0.0004 | 0.0005 | 0.3400 | 14 | 88444752 |
| rs17293632 | T | C | 0.2364 | 0.1072 | 0.0116 | 2.71E-20 | T | C | 0.2358 | 0.0006 | 0.0003 | 0.0740 | 15 | 67442596 |
| rs11641184 | A | C | 0.4762 | 0.0773 | 0.0100 | 1.09E-14 | A | C | 0.4802 | -0.0003 | 0.0003 | 0.3000 | 16 | 11704651 |
| rs12599586 | A | G | 0.7257 | -0.0608 | 0.0110 | 3.57E-08 | A | G | 0.7283 | 0.0003 | 0.0003 | 0.3700 | 16 | 51031876 |
| rs12928665 | G | A | 0.2448 | 0.0653 | 0.0115 | 1.29E-08 | G | A | 0.2442 | 0.0001 | 0.0003 | 0.8100 | 16 | 10971474 |
| rs1528602 | C | T | 0.2620 | -0.0624 | 0.0111 | 2.19E-08 | C | T | 0.2616 | -0.0002 | 0.0003 | 0.4800 | 16 | 50999201 |
| rs1990623 | G | A | 0.1543 | 0.1070 | 0.0133 | 1.06E-15 | G | A | 0.1501 | -0.0005 | 0.0004 | 0.2400 | 16 | 50565970 |
| rs2270395 | T | C | 0.7616 | 0.0778 | 0.0119 | 5.17E-11 | T | C | 0.7727 | 0.0004 | 0.0003 | 0.2700 | 16 | 50846832 |
| rs4643314 | T | C | 0.4978 | 0.0890 | 0.0098 | 1.03E-19 | T | C | 0.4879 | 0.0000 | 0.0003 | 0.9600 | 16 | 50375955 |
| rs7194886 | T | C | 0.4357 | -0.1260 | 0.0100 | 2.53E-36 | T | C | 0.4185 | 0.0003 | 0.0003 | 0.2800 | 16 | 50725193 |
| rs7404095 | C | T | 0.5796 | 0.0557 | 0.0101 | 3.10E-08 | C | T | 0.5817 | -0.0002 | 0.0003 | 0.5100 | 16 | 23864590 |
| rs8055876 | A | G | 0.1078 | 0.1023 | 0.0161 | 2.02E-10 | A | G | 0.1250 | -0.0005 | 0.0004 | 0.2300 | 16 | 11042394 |
| rs17679361 | C | T | 0.0214 | 0.1917 | 0.0330 | 6.18E-09 | C | T | 0.0247 | 0.0006 | 0.0009 | 0.4800 | 17 | 38412542 |
| rs17780256 | C | A | 0.1927 | -0.0834 | 0.0126 | 3.19E-11 | C | A | 0.1830 | 0.0005 | 0.0004 | 0.1800 | 17 | 70642923 |
| rs2945412 | A | G | 0.5866 | 0.0563 | 0.0101 | 2.77E-08 | A | G | 0.5860 | -0.0004 | 0.0003 | 0.1800 | 17 | 25843643 |
| rs3853824 | C | T | 0.6385 | 0.0640 | 0.0104 | 7.70E-10 | C | T | 0.6580 | 0.0004 | 0.0003 | 0.1800 | 17 | 54880993 |
| rs4795397 | G | A | 0.4713 | 0.1383 | 0.0100 | 8.30E-44 | G | A | 0.4739 | -0.0007 | 0.0003 | 0.0150 | 17 | 38027400 |
| rs9889296 | A | G | 0.2723 | -0.1050 | 0.0113 | 1.35E-20 | A | G | 0.2753 | 0.0000 | 0.0003 | 0.9400 | 17 | 32579531 |
| rs67643815 | T | G | 0.5342 | -0.0629 | 0.0102 | 6.42E-10 | T | G | 0.5324 | 0.0000 | 0.0003 | 0.9800 | 18 | 67561508 |
| rs7236492 | T | C | 0.1537 | -0.0803 | 0.0142 | 1.45E-08 | T | C | 0.1719 | 0.0000 | 0.0004 | 0.9200 | 18 | 77220616 |
| rs7240004 | G | A | 0.3795 | -0.0665 | 0.0103 | 1.01E-10 | G | A | 0.3688 | 0.0001 | 0.0003 | 0.6300 | 18 | 46395022 |
| rs12720356 | C | A | 0.0857 | 0.1483 | 0.0182 | 4.13E-16 | C | A | 0.0969 | 0.0000 | 0.0005 | 0.9300 | 19 | 10469975 |
| rs17694108 | A | G | 0.2797 | 0.0858 | 0.0111 | 1.21E-14 | A | G | 0.2760 | -0.0002 | 0.0003 | 0.6300 | 19 | 33731551 |
| rs2024092 | A | G | 0.2162 | 0.1068 | 0.0121 | 1.12E-18 | A | G | 0.2009 | -0.0007 | 0.0003 | 0.0420 | 19 | 1124031 |
| rs35164067 | A | G | 0.2039 | -0.1175 | 0.0127 | 2.66E-20 | A | G | 0.2018 | 0.0003 | 0.0003 | 0.4300 | 19 | 10525181 |
| rs752508 | A | G | 0.2833 | 0.0729 | 0.0116 | 2.88E-10 | A | G | 0.2924 | -0.0002 | 0.0003 | 0.4200 | 19 | 1168753 |
| rs259964 | G | A | 0.5414 | -0.0675 | 0.0098 | 6.93E-12 | G | A | 0.5454 | 0.0000 | 0.0003 | 0.9900 | 20 | 57824309 |
| rs6074022 | T | C | 0.7497 | -0.0743 | 0.0114 | 8.32E-11 | T | C | 0.7546 | -0.0001 | 0.0003 | 0.8700 | 20 | 44740196 |
| rs6111031 | T | C | 0.1591 | -0.2641 | 0.0148 | 1.23E-71 | T | C | 0.1215 | 0.0001 | 0.0004 | 0.8500 | 20 | 1682037 |
| rs1297258 | T | C | 0.4249 | -0.1145 | 0.0101 | 5.38E-30 | T | C | 0.4304 | 0.0001 | 0.0003 | 0.8000 | 21 | 16806709 |
| rs2284553 | G | A | 0.5904 | 0.0595 | 0.0102 | 4.82E-09 | G | A | 0.5897 | -0.0002 | 0.0003 | 0.5100 | 21 | 34776695 |
| rs8127691 | C | T | 0.6132 | -0.1143 | 0.0101 | 8.98E-30 | C | T | 0.6111 | -0.0002 | 0.0003 | 0.4100 | 21 | 45614860 |
| rs2143178 | C | T | 0.1658 | -0.1767 | 0.0137 | 4.80E-38 | C | T | 0.1646 | 0.0000 | 0.0004 | 0.9500 | 22 | 39660829 |

Abbreviation: EA, Effect Allele; OA, Other Allele; EAF, effect allele frequency; SE, standard error; SNP, single nucleotide polymorphism; Chr, Chromosome

**Supplemental Table S3.** **Characteristics of the SNPs used in the Mendelian randomization analysis of the effects of inflammantary bowel disease on colorectal cancer risk**

| SNP | Effects on inflammantary bowel disease | | | | | | Effects on colorectal cancer | | | | | | Chr | Position |
| --- | --- | --- | --- | --- | --- | --- | --- | --- | --- | --- | --- | --- | --- | --- |
|  | EA | OA | EAF | Beta | SE | p-val | EA | OA | EAF | Beta | SE | p-val |  |  |
| rs10494343 | C | T | 0.2067 | 0.0860 | 0.0152 | 1.68E-08 | C | T | 0.2090 | 0.0002 | 0.0003 | 0.6200 | 1 | 160402539 |
| rs10799838 | C | T | 0.7693 | -0.1289 | 0.0144 | 2.87E-19 | C | T | 0.7784 | 0.0006 | 0.0003 | 0.0850 | 1 | 20135822 |
| rs11578752 | T | C | 0.6650 | 0.0816 | 0.0137 | 2.39E-09 | T | C | 0.6650 | -0.0003 | 0.0003 | 0.2800 | 1 | 160845489 |
| rs12142199 | A | G | 0.8026 | -0.0983 | 0.0159 | 6.72E-10 | A | G | 0.7919 | -0.0004 | 0.0003 | 0.2700 | 1 | 1249187 |
| rs2488401 | T | C | 0.2040 | 0.0932 | 0.0153 | 1.12E-09 | T | C | 0.2078 | 0.0001 | 0.0003 | 0.8200 | 1 | 197781198 |
| rs2816958 | G | A | 0.8852 | 0.1808 | 0.0205 | 1.14E-18 | G | A | 0.8868 | 0.0005 | 0.0004 | 0.2200 | 1 | 200101920 |
| rs35223180 | T | G | 0.1791 | -0.1410 | 0.0176 | 1.04E-15 | T | G | 0.1794 | 0.0003 | 0.0004 | 0.4400 | 1 | 8185902 |
| rs4845140 | T | C | 0.0412 | -0.2457 | 0.0341 | 6.25E-13 | T | C | 0.0428 | 0.0008 | 0.0007 | 0.2300 | 1 | 206970470 |
| rs4845604 | A | G | 0.1483 | -0.1637 | 0.0186 | 1.20E-18 | A | G | 0.1462 | 0.0003 | 0.0004 | 0.4800 | 1 | 151801680 |
| rs6700241 | G | A | 0.2154 | 0.0868 | 0.0155 | 2.30E-08 | G | A | 0.2011 | -0.0004 | 0.0004 | 0.2300 | 1 | 161500975 |
| rs10185424 | G | T | 0.5396 | -0.0966 | 0.0126 | 1.47E-14 | G | T | 0.5350 | -0.0002 | 0.0003 | 0.4500 | 2 | 102662888 |
| rs11676348 | T | C | 0.4763 | 0.0744 | 0.0124 | 2.08E-09 | T | C | 0.4822 | -0.0007 | 0.0003 | 0.0110 | 2 | 219010146 |
| rs1517352 | C | A | 0.6048 | 0.0778 | 0.0130 | 2.10E-09 | C | A | 0.6259 | -0.0005 | 0.0003 | 0.0890 | 2 | 191931464 |
| rs4973341 | T | C | 0.6627 | 0.0735 | 0.0131 | 2.25E-08 | T | C | 0.6641 | 0.0006 | 0.0003 | 0.0490 | 2 | 228660362 |
| rs9941524 | G | A | 0.4559 | 0.0977 | 0.0128 | 2.15E-14 | G | A | 0.4327 | 0.0009 | 0.0003 | 0.0015 | 2 | 199499443 |
| rs3774937 | C | T | 0.3257 | 0.0993 | 0.0132 | 4.61E-14 | C | T | 0.3476 | 0.0003 | 0.0003 | 0.3600 | 4 | 103434253 |
| rs17800987 | G | A | 0.0855 | 0.1242 | 0.0215 | 7.99E-09 | G | A | 0.0819 | -0.0005 | 0.0005 | 0.3800 | 5 | 150323428 |
| rs2070729 | A | C | 0.4382 | -0.0828 | 0.0126 | 4.59E-11 | A | C | 0.4401 | -0.0002 | 0.0003 | 0.4800 | 5 | 131819921 |
| rs254562 | G | A | 0.4051 | 0.0796 | 0.0126 | 2.81E-10 | G | A | 0.4093 | 0.0008 | 0.0003 | 0.0033 | 5 | 134441457 |
| rs272882 | T | G | 0.6733 | 0.1459 | 0.0139 | 6.67E-26 | T | G | 0.7062 | 0.0008 | 0.0003 | 0.0098 | 5 | 131669161 |
| rs3776414 | G | T | 0.3756 | 0.0705 | 0.0128 | 4.10E-08 | G | T | 0.3542 | 0.0002 | 0.0003 | 0.5800 | 5 | 10752315 |
| rs395157 | T | C | 0.4842 | 0.0854 | 0.0125 | 8.77E-12 | T | C | 0.4901 | -0.0004 | 0.0003 | 0.1800 | 5 | 38867732 |
| rs56167332 | A | C | 0.3375 | 0.1414 | 0.0132 | 7.27E-27 | A | C | 0.3450 | 0.0000 | 0.0003 | 0.9900 | 5 | 158827769 |
| rs7711427 | C | A | 0.6130 | 0.0889 | 0.0128 | 3.67E-12 | C | A | 0.6046 | -0.0001 | 0.0003 | 0.7200 | 5 | 40414886 |
| rs4946717 | T | C | 0.5097 | -0.0751 | 0.0126 | 2.35E-09 | T | C | 0.5047 | -0.0001 | 0.0003 | 0.6000 | 6 | 106474749 |
| rs79823250 | T | C | 0.1318 | 0.1030 | 0.0182 | 1.52E-08 | T | C | 0.1390 | 0.0001 | 0.0004 | 0.7800 | 6 | 106579725 |
| rs1077773 | A | G | 0.5238 | 0.0721 | 0.0124 | 5.96E-09 | A | G | 0.5331 | 0.0002 | 0.0003 | 0.4600 | 7 | 17443199 |
| rs12718244 | A | G | 0.4081 | 0.0718 | 0.0127 | 1.41E-08 | A | G | 0.4245 | 0.0003 | 0.0003 | 0.2300 | 7 | 50175654 |
| rs17552904 | T | G | 0.2605 | 0.0830 | 0.0141 | 4.25E-09 | T | G | 0.2549 | 0.0004 | 0.0003 | 0.2100 | 7 | 50318308 |
| rs2108225 | A | G | 0.4385 | -0.1147 | 0.0126 | 9.87E-20 | A | G | 0.4236 | -0.0003 | 0.0003 | 0.3400 | 7 | 107453103 |
| rs2395022 | C | A | 0.9589 | -0.1839 | 0.0292 | 2.88E-10 | C | A | 0.9582 | 0.0004 | 0.0007 | 0.6100 | 7 | 98750379 |
| rs4728142 | A | G | 0.4390 | 0.0970 | 0.0127 | 1.92E-14 | A | G | 0.4483 | -0.0006 | 0.0003 | 0.0370 | 7 | 128573967 |
| rs76546301 | A | G | 0.0182 | 0.2650 | 0.0410 | 1.05E-10 | A | G | 0.0182 | 0.0016 | 0.0010 | 0.1300 | 7 | 50498389 |
| rs13255292 | T | C | 0.3284 | -0.0755 | 0.0137 | 3.82E-08 | T | C | 0.3234 | -0.0002 | 0.0003 | 0.5900 | 8 | 129076573 |
| rs4366152 | C | T | 0.6800 | 0.1204 | 0.0136 | 7.79E-19 | C | T | 0.6773 | -0.0004 | 0.0003 | 0.1800 | 9 | 117564875 |
| rs4743820 | T | C | 0.7019 | 0.0809 | 0.0138 | 4.05E-09 | T | C | 0.7005 | -0.0002 | 0.0003 | 0.4400 | 9 | 93933158 |
| rs10761659 | G | A | 0.5399 | 0.1173 | 0.0126 | 1.50E-20 | G | A | 0.5389 | 0.0001 | 0.0003 | 0.7400 | 10 | 64445564 |
| rs2274351 | T | C | 0.5373 | 0.0711 | 0.0130 | 4.90E-08 | T | C | 0.5431 | 0.0000 | 0.0003 | 0.9700 | 10 | 104264107 |
| rs4747886 | T | C | 0.4081 | 0.0738 | 0.0129 | 9.58E-09 | T | C | 0.4182 | -0.0004 | 0.0003 | 0.1300 | 10 | 6176166 |
| rs59418206 | A | G | 0.3508 | 0.0736 | 0.0130 | 1.45E-08 | A | G | 0.3437 | 0.0002 | 0.0003 | 0.4600 | 10 | 35331624 |
| rs11230563 | T | C | 0.3480 | -0.0751 | 0.0134 | 1.90E-08 | T | C | 0.3419 | 0.0000 | 0.0003 | 0.9400 | 11 | 60776209 |
| rs12796489 | A | C | 0.0229 | -0.6765 | 0.0560 | 1.22E-33 | A | C | 0.0097 | -0.0015 | 0.0014 | 0.3000 | 11 | 3059360 |
| rs483905 | A | G | 0.2890 | 0.0850 | 0.0135 | 3.16E-10 | A | G | 0.2971 | 0.0001 | 0.0003 | 0.7100 | 11 | 96023427 |
| rs661054 | G | A | 0.3408 | -0.1249 | 0.0136 | 3.18E-20 | G | A | 0.3293 | 0.0008 | 0.0003 | 0.0055 | 11 | 114430410 |
| rs12318183 | A | C | 0.3854 | 0.1622 | 0.0127 | 1.44E-37 | A | C | 0.3882 | -0.0001 | 0.0003 | 0.6100 | 12 | 68503836 |
| rs76904798 | T | C | 0.1368 | 0.1046 | 0.0176 | 2.78E-09 | T | C | 0.1445 | -0.0004 | 0.0004 | 0.3100 | 12 | 40614434 |
| rs941823 | C | T | 0.7509 | 0.1087 | 0.0147 | 1.39E-13 | C | T | 0.7456 | -0.0007 | 0.0003 | 0.0210 | 13 | 41013977 |
| rs55808324 | A | G | 0.0932 | 0.1272 | 0.0210 | 1.47E-09 | A | G | 0.0986 | 0.0004 | 0.0005 | 0.3400 | 14 | 88444752 |
| rs11641184 | A | C | 0.4762 | 0.0780 | 0.0125 | 4.24E-10 | A | C | 0.4802 | -0.0003 | 0.0003 | 0.3000 | 16 | 11704651 |
| rs7404095 | C | T | 0.5796 | 0.0718 | 0.0127 | 1.52E-08 | C | T | 0.5817 | -0.0002 | 0.0003 | 0.5100 | 16 | 23864590 |
| rs79045992 | A | G | 0.1033 | 0.1181 | 0.0208 | 1.43E-08 | A | G | 0.1084 | 0.0006 | 0.0005 | 0.1800 | 16 | 68518992 |
| rs17679361 | C | T | 0.0214 | 0.2472 | 0.0401 | 6.80E-10 | C | T | 0.0247 | 0.0006 | 0.0009 | 0.4800 | 17 | 38412542 |
| rs17736589 | G | A | 0.2075 | 0.0823 | 0.0150 | 4.34E-08 | G | A | 0.1975 | 0.0001 | 0.0004 | 0.8800 | 17 | 76737118 |
| rs17780256 | C | A | 0.1927 | -0.1154 | 0.0160 | 6.13E-13 | C | A | 0.1830 | 0.0005 | 0.0004 | 0.1800 | 17 | 70642923 |
| rs4795397 | G | A | 0.4713 | 0.1399 | 0.0126 | 1.01E-28 | G | A | 0.4739 | -0.0007 | 0.0003 | 0.0150 | 17 | 38027400 |
| rs9891119 | C | A | 0.3536 | -0.0895 | 0.0133 | 1.72E-11 | C | A | 0.3497 | 0.0001 | 0.0003 | 0.6300 | 17 | 40507980 |
| rs9911533 | T | C | 0.6161 | 0.0766 | 0.0132 | 7.34E-09 | T | C | 0.6351 | 0.0001 | 0.0003 | 0.7500 | 17 | 38775476 |
| rs16939788 | T | C | 0.2459 | 0.0808 | 0.0146 | 3.46E-08 | T | C | 0.2548 | 0.0001 | 0.0003 | 0.7300 | 18 | 12744692 |
| rs7240004 | G | A | 0.3795 | -0.0824 | 0.0130 | 2.50E-10 | G | A | 0.3688 | 0.0001 | 0.0003 | 0.6300 | 18 | 46395022 |
| rs11083840 | G | T | 0.4025 | 0.0691 | 0.0125 | 3.41E-08 | G | T | 0.4084 | -0.0002 | 0.0003 | 0.4500 | 19 | 47119910 |
| rs12720356 | C | A | 0.0857 | 0.1533 | 0.0228 | 1.67E-11 | C | A | 0.0969 | 0.0000 | 0.0005 | 0.9300 | 19 | 10469975 |
| rs17694108 | A | G | 0.2797 | 0.0958 | 0.0139 | 6.17E-12 | A | G | 0.2760 | -0.0002 | 0.0003 | 0.6300 | 19 | 33731551 |
| rs17771967 | G | A | 0.4240 | 0.0705 | 0.0129 | 4.50E-08 | G | A | 0.4206 | -0.0002 | 0.0003 | 0.4100 | 19 | 55380214 |
| rs4812833 | A | G | 0.5188 | 0.1033 | 0.0126 | 1.87E-16 | A | G | 0.5164 | -0.0004 | 0.0003 | 0.1800 | 20 | 43068996 |
| rs6088747 | T | G | 0.5626 | 0.0739 | 0.0125 | 3.82E-09 | T | G | 0.5598 | -0.0005 | 0.0003 | 0.0930 | 20 | 33754604 |
| rs6111031 | T | C | 0.1591 | -0.2609 | 0.0191 | 1.33E-42 | T | C | 0.1215 | 0.0001 | 0.0004 | 0.8500 | 20 | 1682037 |
| rs1297256 | T | C | 0.4249 | -0.1011 | 0.0127 | 2.10E-15 | T | C | 0.4309 | 0.0001 | 0.0003 | 0.7800 | 21 | 16805676 |
| rs7282490 | A | G | 0.6066 | -0.1044 | 0.0127 | 2.06E-16 | A | G | 0.6056 | -0.0002 | 0.0003 | 0.4500 | 21 | 45615741 |
| rs1076137 | G | A | 0.7293 | -0.0823 | 0.0141 | 5.18E-09 | G | A | 0.7367 | 0.0001 | 0.0003 | 0.7300 | 22 | 30223991 |
| rs9611131 | C | T | 0.1477 | -0.1427 | 0.0182 | 3.84E-15 | C | T | 0.1462 | 0.0000 | 0.0004 | 0.9700 | 22 | 39662480 |

Abbreviation: EA, Effect Allele; OA, Other Allele; EAF, effect allele frequency; SE, standard error; SNP, single nucleotide polymorphism; Chr, Chromosome

**Supplemental Table S4.** **Characteristics of the SNPs used in the Mendelian randomization analysis of the effects of colorectal cancer on crohn’s disease risk**

| SNP | Effects on colorectal cancer | | | | | | Effects on crohn’s disease | | | | | | Chr | Position |
| --- | --- | --- | --- | --- | --- | --- | --- | --- | --- | --- | --- | --- | --- | --- |
|  | EA | OA | EAF | Beta | SE | p-val | EA | OA | EAF | Beta | SE | p-val |  |  |
| rs16892766 | C | A | 0.0795 | 0.0031 | 0.0005 | 3.20E-09 | C | A | 0.0812 | -0.0243 | 0.0218 | 0.2662 | 8 | 117630683 |
| rs6983267 | T | G | 0.4824 | -0.0027 | 0.0003 | 9.40E-22 | T | G | 0.4830 | 0.0075 | 0.0125 | 0.5453 | 8 | 128413305 |
| rs3087967 | C | T | 0.6992 | -0.0021 | 0.0003 | 1.10E-11 | C | T | 0.7201 | -0.0281 | 0.0139 | 0.0434 | 11 | 111171709 |
| rs4939827 | C | T | 0.4780 | -0.0024 | 0.0003 | 3.60E-17 | C | T | 0.4878 | 0.0004 | 0.0126 | 0.9743 | 18 | 46453463 |

Abbreviation: EA, Effect Allele; OA, Other Allele; EAF, effect allele frequency; SE, standard error; SNP, single nucleotide polymorphism; Chr, Chromosome

**Supplemental Table S5.** **Characteristics of the SNPs used in the Mendelian randomization analysis of the effects of colorectal cancer on ulcerative colitis risk**

| SNP | Effects on colorectal cancer | | | | | | Effects on ulcerative colitis | | | | | | Chr | Position |
| --- | --- | --- | --- | --- | --- | --- | --- | --- | --- | --- | --- | --- | --- | --- |
|  | EA | OA | EAF | Beta | SE | p-val | EA | OA | EAF | Beta | SE | p-val |  |  |
| rs16892766 | C | A | 0.0795 | 0.0031 | 0.0005 | 3.20E-09 | C | A | 0.0812 | -0.0119 | 0.0181 | 0.5112 | 8 | 117630683 |
| rs6983267 | T | G | 0.4824 | -0.0027 | 0.0003 | 9.40E-22 | T | G | 0.4830 | 0.0151 | 0.0103 | 0.1428 | 8 | 128413305 |
| rs3087967 | C | T | 0.6992 | -0.0021 | 0.0003 | 1.10E-11 | C | T | 0.7201 | -0.0265 | 0.0115 | 0.0213 | 11 | 111171709 |
| rs4939827 | C | T | 0.4780 | -0.0024 | 0.0003 | 3.60E-17 | C | T | 0.4878 | 0.0073 | 0.0105 | 0.4834 | 18 | 46453463 |

Abbreviation: EA, Effect Allele; OA, Other Allele; EAF, effect allele frequency; SE, standard error; SNP, single nucleotide polymorphism; Chr, Chromosome

**Supplemental Table S6.** **Characteristics of the SNPs used in the Mendelian randomization analysis of the effects of colorectal cancer on inflammantary bowel disease risk**

| SNP | Effects on colorectal cancer | | | | | | Effects on inflammantary bowel disease | | | | | | Chr | Position |
| --- | --- | --- | --- | --- | --- | --- | --- | --- | --- | --- | --- | --- | --- | --- |
|  | EA | OA | EAF | Beta | SE | p-val | EA | OA | EAF | Beta | SE | p-val |  |  |
| rs16892766 | C | A | 0.0795 | 0.0031 | 0.0005 | 3.20E-09 | C | A | 0.0812 | -0.0064 | 0.0228 | 0.7775 | 8 | 117630683 |
| rs6983267 | T | G | 0.4824 | -0.0027 | 0.0003 | 9.40E-22 | T | G | 0.4830 | 0.0168 | 0.0129 | 0.1916 | 8 | 128413305 |
| rs3087967 | C | T | 0.6992 | -0.0021 | 0.0003 | 1.10E-11 | C | T | 0.7201 | -0.0304 | 0.0143 | 0.0338 | 11 | 111171709 |
| rs4939827 | C | T | 0.4780 | -0.0024 | 0.0003 | 3.60E-17 | C | T | 0.4878 | 0.0061 | 0.0130 | 0.6421 | 18 | 46453463 |

Abbreviation: EA, Effect Allele; OA, Other Allele; EAF, effect allele frequency; SE, standard error; SNP, single nucleotide polymorphism; Chr, Chromosome

**Supplemental Table S7. Associations of the SNPs with potential confounding factors removed in the Mendelian randomization analysis of crohn’s disease on colorectal cancer risk.**

| SNP | Chr | Position | EA | OA | Association with crohn’s disease | | | | Association with confunding factors | | | |
| --- | --- | --- | --- | --- | --- | --- | --- | --- | --- | --- | --- | --- |
|  |  |  |  |  | Beta | SE | P-val | F-Statistic | Beta | SE | P-val | trait |
| rs26528 | 16 | 28517709 | C | T | 0.1198 | 0.0122 | 1.29E-22 | 95.7654 | 0.1115 | 0.0192 | 6.27E-09 | Body mass index |
| rs17391694 | 1 | 78623626 | T | C | -0.1191 | 0.0200 | 2.62E-09 | 35.4492 | 0.1576 | 0.0284 | 2.88E-08 | Body mass index |
| rs568617 | 11 | 65653242 | T | C | 0.0851 | 0.0151 | 1.75E-08 | 31.7550 | 0.0224 | 0.0030 | 8.12E-14 | Body mass index |
| rs6738825 | 2 | 198896895 | G | A | -0.0748 | 0.0119 | 2.86E-10 | 39.7652 | 0.0131 | 0.0024 | 4.75E-08 | Body mass index |
| rs13109404 | 4 | 102896591 | G | T | 0.1831 | 0.0236 | 8.56E-15 | 60.2026 | -0.0515 | 0.0048 | 1.14E-26 | Calcium |
| rs974801 | 4 | 106071064 | G | A | -0.0700 | 0.0123 | 1.27E-08 | 32.3788 | 0.0173 | 0.0026 | 1.86E-11 | Calcium |
| rs4976646 | 5 | 176788570 | C | T | 0.0696 | 0.0126 | 3.47E-08 | 30.4279 | 0.0182 | 0.0026 | 5.20E-12 | Calcium |
| rs6738490 | 2 | 234161583 | C | T | 0.2262 | 0.0121 | 4.26E-78 | 349.9869 | 0.0276 | 0.0025 | 1.05E-28 | Calcium |
| rs2230365 | 6 | 31525448 | T | C | 0.1068 | 0.0161 | 3.01E-11 | 44.1689 | -0.7089 | 0.0307 | 7.11E-118 | Celiac disease |
| rs4151651 | 6 | 31915614 | A | G | 0.2615 | 0.0315 | 9.79E-17 | 69.0125 | -0.7705 | 0.0628 | 1.27E-34 | Celiac disease |
| rs449242 | 6 | 33496714 | G | T | 0.0718 | 0.0131 | 3.88E-08 | 30.2087 | -0.1688 | 0.0197 | 1.13E-17 | Celiac disease |
| rs7608910 | 2 | 61204856 | G | A | 0.1206 | 0.0121 | 2.95E-23 | 98.6930 | 0.1519 | 0.0189 | 9.66E-16 | Celiac disease |
| rs212388 | 6 | 159490436 | T | C | -0.1024 | 0.0124 | 1.80E-16 | 67.8156 | -0.1222 | 0.0188 | 7.73E-11 | Celiac disease |
| rs11236797 | 11 | 76299649 | A | C | 0.1807 | 0.0121 | 8.54E-51 | 224.6990 | 0.1539 | 0.0248 | 5.41E-10 | Celiac disease |
| rs3024505 | 1 | 206939904 | A | G | 0.1653 | 0.0160 | 3.95E-25 | 107.2390 | 0.2343 | 0.0402 | 5.33E-09 | Celiac disease |
| rs303429 | 10 | 30708441 | T | C | 0.0763 | 0.0124 | 8.38E-10 | 37.6707 | -0.0153 | 0.0025 | 4.54E-10 | C-reactive protein |
| rs4802307 | 19 | 46849806 | T | G | -0.0898 | 0.0132 | 9.04E-12 | 46.5259 | 0.0181 | 0.0026 | 2.84E-12 | C-reactive protein |
| rs1292053 | 17 | 57963537 | G | A | 0.0912 | 0.0119 | 1.75E-14 | 58.7980 | -0.0168 | 0.0024 | 3.21E-12 | C-reactive protein |
| rs6062496 | 20 | 62329099 | A | G | 0.1201 | 0.0124 | 3.82E-22 | 93.6247 | 0.0187 | 0.0024 | 6.89E-15 | C-reactive protein |
| rs11793497 | 9 | 139271850 | G | A | 0.1689 | 0.0122 | 9.80E-44 | 192.3405 | 0.0648 | 0.0107 | 1.23E-09 | C-reactive protein |
| rs61839660 | 10 | 6094697 | T | C | 0.1482 | 0.0203 | 3.19E-13 | 53.0866 | -0.4719 | 0.0360 | 2.84E-39 | Diabetes |
| rs714027 | 22 | 30577771 | G | A | -0.0895 | 0.0123 | 3.51E-13 | 52.8994 | 0.1310 | 0.0190 | 5.40E-12 | Diabetes |
| rs72727394 | 15 | 38847022 | T | C | 0.1032 | 0.0150 | 5.28E-12 | 47.5795 | 0.1380 | 0.0220 | 3.55E-10 | Diabetes |
| rs1847472 | 6 | 90973159 | A | C | -0.0854 | 0.0132 | 1.09E-10 | 41.6538 | 0.1150 | 0.0190 | 1.42E-09 | Diabetes |
| rs3184504 | 12 | 111884608 | C | T | -0.0677 | 0.0120 | 1.71E-08 | 31.7988 | -0.2661 | 0.0190 | 1.56E-44 | Diabetes |
| rs2476601 | 1 | 114377568 | G | A | 0.1851 | 0.0220 | 3.83E-17 | 70.8624 | -0.6360 | 0.0270 | 1.10E-122 | Diabetes |
| rs6827756 | 4 | 123184411 | C | T | -0.0790 | 0.0126 | 3.27E-10 | 39.5060 | -0.1310 | 0.0190 | 5.40E-12 | Diabetes |
| rs438475 | 6 | 32186245 | A | G | 0.1591 | 0.0173 | 3.42E-20 | 84.7328 | 0.6627 | 0.0575 | 1.09E-30 | Diabetes |
| rs516246 | 19 | 49206172 | T | C | 0.1146 | 0.0123 | 1.33E-20 | 86.6010 | 0.1946 | 0.0339 | 9.36E-09 | Diabetes |
| rs6908425 | 6 | 20728731 | C | T | 0.1040 | 0.0150 | 4.81E-12 | 47.7641 | 0.0911 | 0.0085 | 8.41E-27 | Diabetes |
| rs10276381 | 7 | 28190121 | T | C | 0.1159 | 0.0188 | 6.75E-10 | 38.0913 | 0.0713 | 0.0110 | 8.83E-11 | Diabetes |
| rs6915823 | 6 | 30073430 | T | C | 0.1723 | 0.0304 | 1.46E-08 | 32.1108 | 0.1294 | 0.0203 | 1.84E-10 | Diabetes |
| rs2143606 | 20 | 42838550 | G | A | -0.0742 | 0.0121 | 8.36E-10 | 37.6732 | -0.0411 | 0.0069 | 2.58E-09 | Diabetes |
| rs1646019 | 16 | 11359680 | T | C | -0.1114 | 0.0134 | 8.62E-17 | 69.2627 | 0.0180 | 0.0025 | 1.44E-12 | Glycated haemoglobin |
| rs12949918 | 17 | 40526273 | C | T | -0.1042 | 0.0124 | 3.47E-17 | 71.0556 | 0.0136 | 0.0023 | 4.75E-09 | Glycated haemoglobin |
| rs77981966 | 2 | 43777964 | T | C | 0.1827 | 0.0222 | 2.19E-16 | 67.4216 | -0.3606 | 0.0289 | 1.30E-35 | Glycated haemoglobin |
| rs102275 | 11 | 61557803 | C | T | 0.0903 | 0.0124 | 3.98E-13 | 52.6546 | -0.0935 | 0.0161 | 5.74E-09 | Glycated haemoglobin |
| rs2641348 | 1 | 120437884 | G | A | -0.1213 | 0.0198 | 9.65E-10 | 37.3938 | 0.1401 | 0.0246 | 1.21E-08 | Glycated haemoglobin |
| rs3094035 | 6 | 30363136 | T | G | -0.1387 | 0.0214 | 8.36E-11 | 42.1718 | -0.6280 | 0.0704 | 2.12E-18 | Glycated haemoglobin |
| rs17622378 | 5 | 131778452 | G | A | 0.1902 | 0.0121 | 7.17E-56 | 247.9762 | -0.0038 | 0.0007 | 2.00E-08 | hypertension |
| rs13407913 | 2 | 25097644 | G | A | 0.1145 | 0.0120 | 9.64E-22 | 91.7905 | 0.1623 | 0.0264 | 8.42E-10 | Obesity |
| rs7775228 | 6 | 32658079 | C | T | 0.1513 | 0.0176 | 8.20E-18 | 73.9042 | -0.4843 | 0.0510 | 2.63E-22 | Primary sclerosing cholangitis |
| rs2836878 | 21 | 40465534 | A | G | -0.1086 | 0.0139 | 4.61E-15 | 61.4209 | -0.2986 | 0.0410 | 8.93E-14 | Primary sclerosing cholangitis |
| rs3197999 | 3 | 49721532 | A | G | 0.1551 | 0.0129 | 2.05E-33 | 145.0905 | 0.2608 | 0.0360 | 2.60E-13 | Primary sclerosing cholangitis |
| rs9264942 | 6 | 31274380 | C | T | 0.1508 | 0.0128 | 6.78E-32 | 138.1428 | 1.1013 | 0.1339 | 1.98E-16 | Psoriasis |
| rs6738394 | 2 | 219110625 | A | G | 0.0777 | 0.0120 | 8.98E-11 | 42.0325 | 0.0116 | 0.0020 | 5.30E-09 | Triglycerides |
| rs11264426 | 1 | 155940948 | G | T | -0.0887 | 0.0139 | 1.50E-10 | 41.0302 | -0.0135 | 0.0022 | 0.00E+00 | Vitamin D |
| rs780094 | 2 | 27741237 | C | T | -0.1165 | 0.0121 | 4.56E-22 | 93.2720 | 0.4320 | 0.0529 | 3.15E-16 | Vitamin D |

Abbreviation: EA, Effect Allele; OA, Other Allele; EAF, effect allele frequency; SE, standard error; SNP, single nucleotide polymorphism; Chr, Chromosome

**Supplemental Table S8. Associations of the SNPs with potential confounding factors removed in the Mendelian randomization analysis of ulcerative colitis on colorectal cancer risk.**

| SNP | Chr | Position | EA | OA | Association with ulcerative colitis | | | | Association with confunding factors | | | |
| --- | --- | --- | --- | --- | --- | --- | --- | --- | --- | --- | --- | --- |
|  |  |  |  |  | Beta | SE | P-val | F-Statistic | Beta | SE | P-val | trait |
| rs62037363 | 16 | 28865042 | C | T | 0.0988 | 0.0103 | 6.36E-22 | 92.6126 | 0.1457 | 0.0193 | 4.53E-14 | Body mass index |
| rs974801 | 4 | 106071064 | G | A | -0.0728 | 0.0101 | 7.07E-13 | 51.5234 | 0.0173 | 0.0026 | 1.86E-11 | Calcium |
| rs4976646 | 5 | 176788570 | C | T | 0.0730 | 0.0105 | 3.23E-12 | 48.5408 | 0.0182 | 0.0026 | 5.20E-12 | Calcium |
| rs6708373 | 2 | 234172846 | G | A | 0.1342 | 0.0099 | 1.43E-41 | 182.4339 | 0.0277 | 0.0025 | 5.79E-29 | Calcium |
| rs2233965 | 6 | 31080899 | G | T | 0.0992 | 0.0131 | 3.04E-14 | 57.7102 | -0.6099 | 0.0285 | 9.93E-102 | Celiac disease |
| rs7738430 | 6 | 31508836 | C | T | 0.2666 | 0.0281 | 2.46E-21 | 89.9398 | -0.7030 | 0.0714 | 7.62E-23 | Celiac disease |
| rs4151651 | 6 | 31915614 | A | G | 0.3864 | 0.0256 | 1.13E-51 | 228.7356 | -0.7705 | 0.0628 | 1.27E-34 | Celiac disease |
| rs3807039 | 6 | 32078373 | C | A | -0.1493 | 0.0169 | 9.67E-19 | 78.1260 | -0.7805 | 0.0364 | 2.72E-102 | Celiac disease |
| rs9273363 | 6 | 32626272 | A | C | -0.1931 | 0.0120 | 3.30E-58 | 258.6960 | 1.4965 | 0.0389 | 9.88E-324 | Celiac disease |
| rs113010081 | 3 | 46457412 | C | T | 0.0922 | 0.0168 | 4.21E-08 | 30.0495 | -0.2021 | 0.0316 | 1.61E-10 | Celiac disease |
| rs7608910 | 2 | 61204856 | G | A | 0.1264 | 0.0100 | 2.60E-36 | 158.3444 | 0.1519 | 0.0189 | 9.66E-16 | Celiac disease |
| rs1250566 | 10 | 81046453 | A | G | -0.1009 | 0.0110 | 4.77E-20 | 84.0708 | -0.1396 | 0.0203 | 6.27E-12 | Celiac disease |
| rs6933404 | 6 | 137959235 | C | T | 0.0958 | 0.0123 | 5.84E-15 | 60.9549 | 0.2445 | 0.0219 | 7.50E-29 | Celiac disease |
| rs7523442 | 1 | 20165971 | T | C | 0.1245 | 0.0099 | 2.76E-36 | 158.2242 | 0.1795 | 0.0290 | 5.90E-10 | Celiac disease |
| rs3024493 | 1 | 206943968 | A | C | 0.1969 | 0.0132 | 1.65E-50 | 223.3919 | 0.2352 | 0.0403 | 5.38E-09 | Celiac disease |
| rs4676410 | 2 | 241563739 | A | G | 0.1096 | 0.0126 | 4.43E-18 | 75.1191 | 0.2019 | 0.0351 | 9.11E-09 | Celiac disease |
| rs34920465 | 1 | 22700351 | G | A | -0.0883 | 0.0138 | 1.49E-10 | 41.0438 | -0.0175 | 0.0031 | 2.57E-08 | C-reactive protein |
| rs2050392 | 10 | 30691503 | A | G | 0.0691 | 0.0103 | 1.87E-11 | 45.0995 | -0.0164 | 0.0025 | 4.16E-11 | C-reactive protein |
| rs4802307 | 19 | 46849806 | T | G | -0.0611 | 0.0108 | 1.72E-08 | 31.7872 | 0.0181 | 0.0026 | 2.84E-12 | C-reactive protein |
| rs913678 | 20 | 48955424 | C | T | -0.0692 | 0.0105 | 5.35E-11 | 43.0462 | 0.0181 | 0.0026 | 1.23E-12 | C-reactive protein |
| rs1292053 | 17 | 57963537 | G | A | 0.0701 | 0.0098 | 9.89E-13 | 50.8662 | -0.0168 | 0.0024 | 3.21E-12 | C-reactive protein |
| rs6062496 | 20 | 62329099 | A | G | 0.1232 | 0.0102 | 2.11E-33 | 145.0378 | 0.0187 | 0.0024 | 6.89E-15 | C-reactive protein |
| rs11793497 | 9 | 139271850 | G | A | 0.1562 | 0.0100 | 1.71E-54 | 241.6582 | 0.0648 | 0.0107 | 1.23E-09 | C-reactive protein |
| rs7547569 | 1 | 67731368 | C | T | -0.6472 | 0.0233 | 1.65E-170 | 774.7674 | -0.3536 | 0.0599 | 3.54E-09 | C-reactive protein |
| rs11236797 | 11 | 76299649 | A | C | 0.1509 | 0.0100 | 9.32E-52 | 229.1100 | 0.1539 | 0.0248 | 5.41E-10 | C-reactive protein |
| rs3806308 | 1 | 20142866 | T | C | -0.0981 | 0.0103 | 1.08E-21 | 91.5588 | -0.0010 | 0.0002 | 3.49E-08 | C-reactive protein |
| rs2847278 | 18 | 12778715 | T | C | -0.1445 | 0.0132 | 8.33E-28 | 119.4551 | -0.1880 | 0.0240 | 4.75E-15 | Diabetes |
| rs1003342 | 22 | 30570022 | G | A | -0.0802 | 0.0102 | 3.30E-15 | 62.0804 | 0.1310 | 0.0190 | 5.40E-12 | Diabetes |
| rs17651741 | 15 | 38869666 | A | G | 0.0702 | 0.0127 | 2.81E-08 | 30.8353 | 0.1310 | 0.0230 | 1.23E-08 | Diabetes |
| rs1847472 | 6 | 90973159 | A | C | -0.0673 | 0.0109 | 6.63E-10 | 38.1273 | 0.1150 | 0.0190 | 1.42E-09 | Diabetes |
| rs3184504 | 12 | 111884608 | C | T | -0.0600 | 0.0099 | 1.29E-09 | 36.8284 | -0.2661 | 0.0190 | 1.56E-44 | Diabetes |
| rs516246 | 19 | 49206172 | T | C | 0.0756 | 0.0102 | 1.15E-13 | 55.0951 | 0.1946 | 0.0339 | 9.36E-09 | Diabetes |
| rs2328546 | 6 | 20657345 | C | T | 0.0940 | 0.0127 | 1.30E-13 | 54.8557 | 0.0693 | 0.0087 | 1.65E-15 | Diabetes |
| rs2143606 | 20 | 42838550 | G | A | -0.0566 | 0.0100 | 1.38E-08 | 32.2109 | -0.0411 | 0.0069 | 2.58E-09 | Diabetes |
| rs367569 | 16 | 11365500 | T | C | -0.0958 | 0.0113 | 1.93E-17 | 72.2185 | 0.0179 | 0.0026 | 2.31E-12 | Glycated haemoglobin |
| rs406113 | 6 | 28483482 | C | A | 0.0578 | 0.0105 | 4.06E-08 | 30.1180 | -0.0147 | 0.0025 | 2.34E-09 | Glycated haemoglobin |
| rs744166 | 17 | 40514201 | G | A | -0.1000 | 0.0102 | 1.14E-22 | 96.0064 | 0.0135 | 0.0023 | 6.01E-09 | Glycated haemoglobin |
| rs1535 | 11 | 61597972 | G | A | 0.0619 | 0.0104 | 2.78E-09 | 35.3289 | -0.0974 | 0.0161 | 1.47E-09 | Glycated haemoglobin |
| rs2497318 | 10 | 94432000 | T | C | -0.0635 | 0.0099 | 1.36E-10 | 41.2234 | 0.1435 | 0.0154 | 1.53E-20 | Glycated haemoglobin |
| rs11745587 | 5 | 131796922 | A | G | -0.1207 | 0.0105 | 1.87E-30 | 131.5579 | 0.0150 | 0.0024 | 4.42E-10 | Glycated haemoglobin |
| rs2974935 | 1 | 155181843 | T | G | 0.0687 | 0.0101 | 8.87E-12 | 46.5638 | 0.0877 | 0.0153 | 1.12E-08 | Glycated haemoglobin |
| rs11677953 | 2 | 219121663 | A | G | 0.0791 | 0.0100 | 2.92E-15 | 62.3183 | -0.0138 | 0.0023 | 4.34E-09 | Glycated haemoglobin |
| rs11229555 | 11 | 58408687 | T | G | -0.0788 | 0.0114 | 5.23E-12 | 47.6005 | -0.0062 | 0.0011 | 4.80E-09 | Hypertension |
| rs13407913 | 2 | 25097644 | G | A | 0.0917 | 0.0099 | 1.69E-20 | 86.1259 | 0.1623 | 0.0264 | 8.42E-10 | Obesity |
| rs2836883 | 21 | 40466744 | A | G | -0.1684 | 0.0115 | 3.38E-48 | 212.7923 | -0.3016 | 0.0410 | 5.40E-14 | Primary sclerosing cholangitis |
| rs9836291 | 3 | 49697459 | A | G | 0.1609 | 0.0105 | 9.61E-53 | 233.6357 | 0.2814 | 0.0310 | 4.51E-20 | Primary sclerosing cholangitis |
| rs7657746 | 4 | 123161619 | G | A | -0.0869 | 0.0118 | 1.83E-13 | 54.1737 | -0.2151 | 0.0360 | 1.79E-09 | Primary sclerosing cholangitis |
| rs2179070 | 6 | 111885752 | C | T | 0.1694 | 0.0197 | 8.54E-18 | 73.8247 | 0.3795 | 0.0528 | 6.31E-13 | Psoriasis |
| rs1990760 | 2 | 163124051 | T | C | -0.0671 | 0.0107 | 3.56E-10 | 39.3391 | 0.1923 | 0.0342 | 1.95E-08 | Psoriasis |
| rs6058869 | 20 | 31348750 | T | C | 0.0557 | 0.0100 | 2.63E-08 | 30.9591 | 0.0208 | 0.0036 | 1.01E-08 | Triglycerides |
| rs780094 | 2 | 27741237 | C | T | -0.0783 | 0.0100 | 3.88E-15 | 61.7582 | 0.4320 | 0.0529 | 3.15E-16 | Vitamin D |
| rs1182188 | 7 | 2869985 | C | T | -0.0659 | 0.0108 | 1.08E-09 | 37.1765 | -0.0156 | 0.0023 | 2.88E-11 | Waist circumference |

Abbreviation: EA, Effect Allele; OA, Other Allele; EAF, effect allele frequency; SE, standard error; SNP, single nucleotide polymorphism; Chr, Chromosome

**Supplemental Table S9. Associations of the SNPs with potential confounding factors removed in the Mendelian randomization analysis of inflammantary bowel disease on colorectal cancer risk.**

| SNP | Chr | Position | EA | OA | Association with inflammantary bowel disease | | | | Association with confunding factors | | | |
| --- | --- | --- | --- | --- | --- | --- | --- | --- | --- | --- | --- | --- |
|  |  |  |  |  | Beta | SE | P-val | F-Statistic | Beta | SE | P-val | trait |
| rs10460566 | 2 | 25483121 | A | G | -0.0818 | 0.0145 | 1.60E-08 | 31.9322 | -0.0115 | 0.0020 | 7.80E-09 | body mass index |
| rs10910092 | 1 | 2501516 | G | A | -0.0864 | 0.0128 | 1.42E-11 | 45.6380 | 0.0108 | 0.0020 | 4.10E-08 | body mass index |
| rs11150589 | 16 | 30482494 | C | T | -0.0799 | 0.0127 | 3.28E-10 | 39.4980 | -0.0127 | 0.0020 | 2.10E-10 | body mass index |
| rs4976646 | 5 | 176788570 | C | T | 0.0788 | 0.0132 | 2.52E-09 | 35.5256 | 0.0182 | 0.0026 | 5.20E-12 | Calcium |
| rs4713240 | 6 | 29710726 | G | A | 0.0927 | 0.0145 | 1.47E-10 | 41.0627 | -0.3048 | 0.0230 | 4.63E-40 | Celiac disease |
| rs17190351 | 6 | 31047448 | A | G | 0.2718 | 0.0402 | 1.43E-11 | 45.6231 | -1.0043 | 0.0745 | 2.02E-41 | Celiac disease |
| rs7738430 | 6 | 31508836 | C | T | 0.3680 | 0.0341 | 3.51E-27 | 116.6019 | -0.7030 | 0.0714 | 7.62E-23 | Celiac disease |
| rs9368696 | 6 | 31554382 | A | G | 0.2481 | 0.0382 | 8.64E-11 | 42.1071 | -0.6763 | 0.0829 | 3.32E-16 | Celiac disease |
| rs9271255 | 6 | 32580357 | T | C | -0.2850 | 0.0138 | 1.31E-94 | 425.8340 | 0.9921 | 0.0258 | 9.88E-324 | Celiac disease |
| rs9272105 | 6 | 32599999 | A | G | 0.1292 | 0.0129 | 1.45E-23 | 100.0945 | 0.6268 | 0.0208 | 6.89E-199 | Celiac disease |
| rs151723 | 6 | 32895359 | G | A | -0.2148 | 0.0376 | 1.08E-08 | 32.6830 | 0.5271 | 0.0433 | 5.09E-34 | Celiac disease |
| rs1431403 | 6 | 33047031 | C | T | -0.1260 | 0.0149 | 2.34E-17 | 71.8354 | 0.6482 | 0.0200 | 1.92E-231 | Celiac disease |
| rs113010081 | 3 | 46457412 | C | T | 0.1271 | 0.0207 | 9.02E-10 | 37.5267 | -0.2021 | 0.0316 | 1.61E-10 | Celiac disease |
| rs7608910 | 2 | 61204856 | G | A | 0.1271 | 0.0127 | 1.25E-23 | 100.3914 | 0.1519 | 0.0189 | 9.66E-16 | Celiac disease |
| rs6920220 | 6 | 138006504 | A | G | 0.1469 | 0.0152 | 4.78E-22 | 93.1779 | 0.2500 | 0.0221 | 9.09E-30 | Celiac disease |
| rs7547569 | 1 | 67731368 | C | T | -0.4957 | 0.0292 | 8.71E-65 | 288.8827 | -0.3536 | 0.0599 | 3.54E-09 | Celiac disease |
| rs61893460 | 11 | 76291154 | A | G | 0.1211 | 0.0125 | 4.60E-22 | 93.2531 | 0.1537 | 0.0248 | 5.75E-10 | Celiac disease |
| rs10748783 | 10 | 101285872 | A | C | -0.1648 | 0.0126 | 7.73E-39 | 169.9142 | -0.1400 | 0.0244 | 9.35E-09 | Celiac disease |
| rs1801274 | 1 | 161479745 | G | A | -0.1709 | 0.0127 | 1.43E-41 | 182.4218 | -0.1390 | 0.0243 | 1.14E-08 | Celiac disease |
| rs3806308 | 1 | 20142866 | T | C | -0.1698 | 0.0130 | 5.56E-39 | 170.5688 | -0.0010 | 0.0002 | 3.49E-08 | Celiac disease |
| rs34920465 | 1 | 22700351 | G | A | -0.1290 | 0.0176 | 2.12E-13 | 53.8931 | -0.0175 | 0.0031 | 2.57E-08 | C-reactive protein |
| rs913678 | 20 | 48955424 | C | T | -0.0758 | 0.0133 | 1.23E-08 | 32.4405 | 0.0181 | 0.0026 | 1.23E-12 | C-reactive protein |
| rs6062496 | 20 | 62329099 | A | G | 0.1139 | 0.0129 | 9.14E-19 | 78.2365 | 0.0187 | 0.0024 | 6.89E-15 | C-reactive protein |
| rs6426833 | 1 | 20171860 | A | G | 0.2324 | 0.0126 | 3.77E-76 | 341.0467 | 0.1897 | 0.0290 | 5.74E-11 | C-reactive protein |
| rs3024493 | 1 | 206943968 | A | C | 0.2263 | 0.0163 | 1.42E-43 | 191.6034 | 0.2352 | 0.0403 | 5.38E-09 | C-reactive protein |
| rs4676410 | 2 | 241563739 | A | G | 0.1420 | 0.0157 | 1.85E-19 | 81.3876 | 0.2019 | 0.0351 | 9.11E-09 | C-reactive protein |
| rs1646019 | 16 | 11359680 | T | C | -0.0772 | 0.0139 | 2.62E-08 | 30.9739 | 0.0180 | 0.0025 | 1.44E-12 | Glycated haemoglobin |
| rs13430791 | 2 | 43481013 | A | G | 0.1059 | 0.0187 | 1.39E-08 | 32.1950 | -0.2678 | 0.0231 | 4.33E-31 | Glycated haemoglobin |
| rs2497318 | 10 | 94432000 | T | C | -0.0714 | 0.0125 | 1.15E-08 | 32.5757 | 0.1435 | 0.0154 | 1.53E-20 | Glycated haemoglobin |
| rs10010325 | 4 | 106106353 | A | C | -0.0830 | 0.0124 | 2.18E-11 | 44.8060 | 0.0126 | 0.0015 | 2.80E-17 | Glycated haemoglobin |
| rs11041476 | 11 | 1875067 | A | G | 0.0735 | 0.0133 | 2.92E-08 | 30.7615 | 0.0561 | 0.0098 | 9.27E-09 | Hypertension |
| rs11229555 | 11 | 58408687 | T | G | -0.0823 | 0.0145 | 1.21E-08 | 32.4633 | -0.0062 | 0.0011 | 4.80E-09 | hypertension |
| rs130067 | 6 | 31118511 | G | T | -0.1298 | 0.0164 | 2.05E-15 | 63.0113 | -0.4233 | 0.0410 | 1.30E-25 | Primary sclerosing cholangitis |
| rs411326 | 6 | 32211317 | T | C | -0.1670 | 0.0156 | 1.06E-26 | 114.4104 | -0.4543 | 0.0420 | 3.42E-28 | Primary sclerosing cholangitis |
| rs2836883 | 21 | 40466744 | A | G | -0.2271 | 0.0147 | 1.47E-53 | 237.3711 | -0.3016 | 0.0410 | 5.40E-14 | Primary sclerosing cholangitis |
| rs9836291 | 3 | 49697459 | A | G | 0.1703 | 0.0132 | 8.20E-38 | 165.2166 | 0.2814 | 0.0310 | 4.51E-20 | Primary sclerosing cholangitis |
| rs13136827 | 4 | 123171318 | C | T | -0.1118 | 0.0176 | 2.35E-10 | 40.1550 | -0.2608 | 0.0440 | 1.10E-09 | Primary sclerosing cholangitis |
| rs34659678 | 6 | 111888540 | T | C | 0.2099 | 0.0251 | 5.95E-17 | 69.9926 | 0.4159 | 0.0537 | 9.02E-15 | Psoriasis |
| rs1990760 | 2 | 163124051 | T | C | -0.0856 | 0.0134 | 1.78E-10 | 40.6936 | 0.1923 | 0.0342 | 1.95E-08 | Psoriasis |
| rs4712520 | 6 | 20640871 | C | T | 0.0930 | 0.0166 | 2.21E-08 | 31.3020 | 0.0598 | 0.0088 | 1.08E-11 | Type 2 diabetes |
| rs1182188 | 7 | 2869985 | C | T | -0.1076 | 0.0138 | 5.03E-15 | 61.2486 | -0.0156 | 0.0023 | 2.88E-11 | Waist circumference |

Abbreviation: EA, Effect Allele; OA, Other Allele; EAF, effect allele frequency; SE, standard error; SNP, single nucleotide polymorphism; Chr, Chromosome

**Supplemental Table S10.** **MR-steiger test results of the SNPs used in the Mendelian randomization analysis of the effects of crohn’s disease on colorectal cancer risk**

| SNP | R^2^ of exposure | R2 of outcome | Steiger direction | Steiger p value |
| --- | --- | --- | --- | --- |
| rs10055349 | 2.32E-03 | 1.59E-05 | TRUE | 3.71E-21 |
| rs10798069 | 6.65E-04 | 1.43E-08 | TRUE | 4.21E-08 |
| rs10800309 | 9.85E-04 | 5.95E-06 | TRUE | 6.19E-10 |
| rs11117431 | 1.59E-03 | 2.08E-07 | TRUE | 3.96E-17 |
| rs11152949 | 2.09E-03 | 1.72E-07 | TRUE | 4.00E-22 |
| rs11159833 | 1.08E-03 | 4.10E-06 | TRUE | 4.78E-11 |
| rs116675765 | 7.29E-04 | 1.06E-06 | TRUE | 2.92E-08 |
| rs11679753 | 8.35E-04 | 4.68E-07 | TRUE | 1.67E-09 |
| rs11691685 | 8.81E-04 | 2.89E-06 | TRUE | 2.26E-09 |
| rs11713774 | 1.15E-03 | 3.15E-08 | TRUE | 5.70E-13 |
| rs12041056 | 1.54E-03 | 5.62E-11 | TRUE | 4.88E-17 |
| rs12194548 | 5.76E-04 | 4.06E-07 | TRUE | 6.08E-07 |
| rs12411259 | 1.84E-03 | 8.94E-07 | TRUE | 3.19E-19 |
| rs1250573 | 2.14E-03 | 6.15E-06 | TRUE | 9.05E-21 |
| rs1267501 | 6.34E-04 | 1.90E-06 | TRUE | 3.70E-07 |
| rs12718244 | 8.35E-04 | 3.83E-06 | TRUE | 8.72E-09 |
| rs12720356 | 8.46E-04 | 2.08E-08 | TRUE | 6.25E-10 |
| rs12796489 | 4.33E-03 | 2.83E-06 | TRUE | 7.71E-43 |
| rs1297258 | 2.09E-03 | 1.72E-07 | TRUE | 3.88E-22 |
| rs13001325 | 1.83E-03 | 1.76E-07 | TRUE | 1.32E-19 |
| rs13204048 | 5.93E-04 | 2.91E-07 | TRUE | 3.65E-07 |
| rs1363907 | 1.28E-03 | 7.01E-06 | TRUE | 1.58E-12 |
| rs1486418 | 6.03E-04 | 4.11E-06 | TRUE | 1.50E-06 |
| rs1517352 | 7.95E-04 | 7.66E-06 | TRUE | 5.53E-08 |
| rs1528602 | 1.39E-03 | 1.33E-06 | TRUE | 1.05E-14 |
| rs1567009 | 1.42E-03 | 1.52E-06 | TRUE | 7.11E-15 |
| rs1569328 | 8.22E-04 | 3.78E-10 | TRUE | 9.35E-10 |
| rs16870166 | 6.21E-04 | 2.21E-06 | TRUE | 5.56E-07 |
| rs16917597 | 6.26E-04 | 1.00E-08 | TRUE | 1.03E-07 |
| rs17293632 | 1.63E-03 | 8.44E-06 | TRUE | 1.24E-15 |
| rs17694108 | 6.74E-04 | 6.08E-07 | TRUE | 7.43E-08 |
| rs181826 | 1.18E-03 | 7.31E-06 | TRUE | 1.27E-11 |
| rs1896707 | 3.82E-03 | 2.46E-06 | TRUE | 5.29E-38 |
| rs1990623 | 3.20E-03 | 3.71E-06 | TRUE | 1.65E-31 |
| rs1990684 | 6.85E-04 | 1.24E-07 | TRUE | 3.48E-08 |
| rs2024092 | 2.04E-03 | 1.09E-05 | TRUE | 3.62E-19 |
| rs2058813 | 1.17E-03 | 3.54E-06 | TRUE | 4.97E-12 |
| rs2153283 | 9.46E-04 | 3.78E-06 | TRUE | 7.45E-10 |
| rs2227551 | 1.01E-03 | 1.02E-06 | TRUE | 5.18E-11 |
| rs224140 | 6.03E-04 | 2.89E-06 | TRUE | 1.04E-06 |
| rs2270395 | 1.42E-03 | 3.23E-06 | TRUE | 1.77E-14 |
| rs2284553 | 1.35E-03 | 1.18E-06 | TRUE | 2.59E-14 |
| rs2395022 | 7.63E-04 | 6.75E-07 | TRUE | 1.04E-08 |
| rs2413583 | 3.00E-03 | 9.27E-12 | TRUE | 1.11E-31 |
| rs2488401 | 1.30E-03 | 1.35E-07 | TRUE | 2.43E-14 |
| rs2538470 | 7.17E-04 | 7.22E-07 | TRUE | 3.03E-08 |
| rs259964 | 6.92E-04 | 3.99E-10 | TRUE | 1.99E-08 |
| rs28999107 | 8.83E-04 | 2.27E-05 | TRUE | 9.92E-08 |
| rs2945412 | 1.62E-03 | 4.69E-06 | TRUE | 4.01E-16 |
| rs3130186 | 6.75E-04 | 1.08E-05 | TRUE | 1.24E-06 |
| rs34779708 | 2.27E-03 | 1.42E-06 | TRUE | 3.42E-23 |
| rs34787213 | 1.29E-03 | 1.09E-06 | TRUE | 9.69E-14 |
| rs34804116 | 1.06E-03 | 1.48E-07 | TRUE | 6.70E-12 |
| rs35164067 | 1.63E-03 | 1.63E-06 | TRUE | 6.11E-17 |
| rs35320439 | 7.19E-04 | 2.41E-07 | TRUE | 1.86E-08 |
| rs36016881 | 7.88E-04 | 3.53E-07 | TRUE | 4.39E-09 |
| rs367254 | 7.40E-04 | 3.31E-06 | TRUE | 5.94E-08 |
| rs3776414 | 1.01E-03 | 7.93E-07 | TRUE | 4.63E-11 |
| rs3801810 | 1.08E-03 | 7.68E-06 | TRUE | 1.24E-10 |
| rs3812609 | 5.92E-04 | 9.93E-08 | TRUE | 2.89E-07 |
| rs3853824 | 8.00E-04 | 4.77E-06 | TRUE | 2.49E-08 |
| rs395157 | 1.30E-03 | 4.72E-06 | TRUE | 5.00E-13 |
| rs41295117 | 8.10E-04 | 1.43E-05 | TRUE | 1.35E-07 |
| rs4643314 | 2.12E-03 | 6.46E-09 | TRUE | 9.34E-23 |
| rs4655709 | 1.47E-03 | 2.46E-06 | TRUE | 4.00E-15 |
| rs4703855 | 5.91E-04 | 1.36E-08 | TRUE | 2.35E-07 |
| rs4767956 | 7.51E-04 | 3.68E-08 | TRUE | 6.16E-09 |
| rs4795397 | 2.33E-03 | 1.54E-05 | TRUE | 2.77E-21 |
| rs4917129 | 1.13E-03 | 1.97E-06 | TRUE | 6.53E-12 |
| rs559928 | 7.56E-04 | 2.04E-07 | TRUE | 7.62E-09 |
| rs56163845 | 8.95E-04 | 1.41E-06 | TRUE | 8.44E-10 |
| rs56167332 | 3.49E-03 | 9.07E-10 | TRUE | 1.67E-36 |
| rs6074022 | 9.42E-04 | 6.66E-08 | TRUE | 7.99E-11 |
| rs6111031 | 4.66E-03 | 9.33E-08 | TRUE | 7.34E-48 |
| rs61959448 | 7.90E-04 | 7.32E-08 | TRUE | 2.72E-09 |
| rs640466 | 7.09E-04 | 6.73E-06 | TRUE | 2.84E-07 |
| rs6456426 | 1.32E-03 | 9.16E-06 | TRUE | 1.21E-12 |
| rs6561151 | 2.06E-03 | 1.64E-06 | TRUE | 4.59E-21 |
| rs6651252 | 1.28E-03 | 7.41E-07 | TRUE | 9.34E-14 |
| rs6740462 | 9.58E-04 | 9.27E-07 | TRUE | 1.48E-10 |
| rs7015630 | 7.23E-04 | 3.13E-06 | TRUE | 8.07E-08 |
| rs7097656 | 1.45E-03 | 1.85E-06 | TRUE | 4.22E-15 |
| rs71624119 | 7.35E-04 | 3.68E-09 | TRUE | 7.59E-09 |
| rs7194886 | 6.66E-03 | 3.05E-06 | TRUE | 1.79E-65 |
| rs7195296 | 8.57E-04 | 9.72E-08 | TRUE | 6.15E-10 |
| rs7236492 | 6.36E-04 | 2.68E-08 | TRUE | 8.65E-08 |
| rs727563 | 7.82E-04 | 1.21E-06 | TRUE | 9.57E-09 |
| rs7438704 | 8.46E-04 | 3.28E-05 | TRUE | 6.01E-07 |
| rs7517847 | 1.38E-02 | 4.15E-08 | TRUE | 1.93E-139 |
| rs752508 | 1.16E-03 | 1.72E-06 | TRUE | 2.51E-12 |
| rs76181804 | 8.01E-04 | 7.77E-06 | TRUE | 5.02E-08 |
| rs76546301 | 6.34E-04 | 6.06E-06 | TRUE | 1.23E-06 |
| rs76606004 | 6.53E-04 | 8.07E-06 | TRUE | 1.22E-06 |
| rs76906269 | 2.18E-03 | 1.20E-06 | TRUE | 1.87E-22 |
| rs7711427 | 7.57E-03 | 3.42E-07 | TRUE | 2.07E-76 |
| rs7758080 | 6.45E-04 | 4.40E-06 | TRUE | 6.52E-07 |
| rs7773324 | 7.17E-04 | 1.40E-06 | TRUE | 4.60E-08 |
| rs7848647 | 2.27E-03 | 4.11E-06 | TRUE | 1.70E-22 |
| rs7969592 | 7.18E-04 | 1.21E-07 | TRUE | 1.62E-08 |
| rs8127691 | 1.97E-03 | 1.77E-06 | TRUE | 3.49E-20 |
| rs915286 | 5.97E-04 | 8.81E-07 | TRUE | 5.19E-07 |
| rs925255 | 1.33E-03 | 4.40E-08 | TRUE | 1.05E-14 |
| rs9457247 | 1.91E-03 | 1.55E-09 | TRUE | 9.96E-21 |
| rs9491892 | 1.36E-03 | 1.46E-05 | TRUE | 1.48E-12 |
| rs9494844 | 7.52E-04 | 1.02E-06 | TRUE | 1.69E-08 |
| rs9554587 | 8.13E-04 | 1.94E-06 | TRUE | 6.93E-09 |
| rs9889296 | 2.07E-03 | 4.20E-08 | TRUE | 3.36E-22 |

**Supplemental Table S11.** **MR-steiger test results of the SNPs used in the Mendelian randomization analysis of the effects of ulcerative colitis on colorectal cancer risk**

| SNP | R^2^ of exposure | R^2^ of outcome | steiger direction | steiger p value |
| --- | --- | --- | --- | --- |
| rs10185424 | 1.24E-03 | 1.53E-06 | TRUE | 2.77E-12 |
| rs10494343 | 6.66E-04 | 6.64E-07 | TRUE | 2.64E-07 |
| rs1076137 | 7.14E-04 | 3.11E-07 | TRUE | 7.12E-08 |
| rs10761659 | 1.81E-03 | 2.91E-07 | TRUE | 5.50E-18 |
| rs1077773 | 7.08E-04 | 1.47E-06 | TRUE | 1.69E-07 |
| rs10799838 | 1.68E-03 | 7.87E-06 | TRUE | 3.40E-15 |
| rs11083840 | 6.38E-04 | 1.50E-06 | TRUE | 7.53E-07 |
| rs11230563 | 6.61E-04 | 1.56E-08 | TRUE | 1.37E-07 |
| rs11578752 | 7.46E-04 | 3.08E-06 | TRUE | 1.42E-07 |
| rs11641184 | 8.16E-04 | 2.80E-06 | TRUE | 3.04E-08 |
| rs11676348 | 7.51E-04 | 1.70E-05 | TRUE | 1.61E-06 |
| rs12142199 | 7.97E-04 | 3.17E-06 | TRUE | 5.08E-08 |
| rs12318183 | 3.43E-03 | 6.91E-07 | TRUE | 1.31E-32 |
| rs12718244 | 6.73E-04 | 3.83E-06 | TRUE | 7.78E-07 |
| rs12720356 | 9.48E-04 | 2.08E-08 | TRUE | 2.75E-10 |
| rs12796489 | 3.05E-03 | 2.83E-06 | TRUE | 2.52E-28 |
| rs1297256 | 1.32E-03 | 2.12E-07 | TRUE | 1.58E-13 |
| rs13255292 | 6.33E-04 | 7.77E-07 | TRUE | 5.75E-07 |
| rs1517352 | 7.51E-04 | 7.66E-06 | TRUE | 3.91E-07 |
| rs16939788 | 6.37E-04 | 3.08E-07 | TRUE | 3.72E-07 |
| rs17552904 | 7.22E-04 | 4.09E-06 | TRUE | 3.10E-07 |
| rs17679361 | 7.97E-04 | 1.33E-06 | TRUE | 2.46E-08 |
| rs17694108 | 9.89E-04 | 6.08E-07 | TRUE | 2.67E-10 |
| rs17736589 | 6.28E-04 | 5.76E-08 | TRUE | 3.22E-07 |
| rs17771967 | 6.26E-04 | 1.77E-06 | TRUE | 1.06E-06 |
| rs17780256 | 1.08E-03 | 4.85E-06 | TRUE | 2.50E-10 |
| rs17800987 | 6.97E-04 | 2.08E-06 | TRUE | 2.78E-07 |
| rs2070729 | 9.07E-04 | 1.32E-06 | TRUE | 2.43E-09 |
| rs2108225 | 1.73E-03 | 2.43E-06 | TRUE | 1.69E-16 |
| rs2274351 | 6.23E-04 | 2.76E-09 | TRUE | 2.92E-07 |
| rs2395022 | 8.32E-04 | 6.75E-07 | TRUE | 7.89E-09 |
| rs2488401 | 7.77E-04 | 1.35E-07 | TRUE | 1.49E-08 |
| rs254562 | 8.33E-04 | 2.29E-05 | TRUE | 7.09E-07 |
| rs272882 | 2.31E-03 | 1.77E-05 | TRUE | 1.47E-19 |
| rs2816958 | 1.63E-03 | 3.95E-06 | TRUE | 2.80E-15 |
| rs35223180 | 1.35E-03 | 1.58E-06 | TRUE | 2.92E-13 |
| rs3774937 | 1.19E-03 | 2.19E-06 | TRUE | 1.04E-11 |
| rs3776414 | 6.30E-04 | 7.93E-07 | TRUE | 6.18E-07 |
| rs395157 | 9.75E-04 | 4.72E-06 | TRUE | 2.20E-09 |
| rs4366152 | 1.64E-03 | 4.74E-06 | TRUE | 2.78E-15 |
| rs4728142 | 1.23E-03 | 1.16E-05 | TRUE | 7.43E-11 |
| rs4743820 | 7.24E-04 | 1.58E-06 | TRUE | 1.27E-07 |
| rs4747886 | 6.89E-04 | 6.19E-06 | TRUE | 9.90E-07 |
| rs4795397 | 2.58E-03 | 1.55E-05 | TRUE | 4.39E-22 |
| rs4812833 | 1.42E-03 | 4.67E-06 | TRUE | 2.72E-13 |
| rs483905 | 8.28E-04 | 3.63E-07 | TRUE | 6.54E-09 |
| rs4845140 | 1.08E-03 | 3.74E-06 | TRUE | 1.77E-10 |
| rs4845604 | 1.62E-03 | 1.30E-06 | TRUE | 7.08E-16 |
| rs4946717 | 7.46E-04 | 7.16E-07 | TRUE | 5.00E-08 |
| rs4973341 | 6.54E-04 | 1.03E-05 | TRUE | 4.07E-06 |
| rs55808324 | 7.65E-04 | 2.41E-06 | TRUE | 7.54E-08 |
| rs56167332 | 2.41E-03 | 9.07E-10 | TRUE | 5.49E-24 |
| rs59418206 | 6.72E-04 | 1.45E-06 | TRUE | 3.56E-07 |
| rs6088747 | 7.27E-04 | 7.49E-06 | TRUE | 6.12E-07 |
| rs6111031 | 3.90E-03 | 9.33E-08 | TRUE | 1.29E-37 |
| rs661054 | 1.77E-03 | 2.04E-05 | TRUE | 9.30E-15 |
| rs6700241 | 6.54E-04 | 3.85E-06 | TRUE | 1.17E-06 |
| rs7240004 | 8.38E-04 | 6.26E-07 | TRUE | 6.72E-09 |
| rs7282490 | 1.41E-03 | 1.53E-06 | TRUE | 7.04E-14 |
| rs7404095 | 6.70E-04 | 1.15E-06 | TRUE | 3.22E-07 |
| rs76546301 | 8.73E-04 | 6.06E-06 | TRUE | 2.42E-08 |
| rs76904798 | 7.40E-04 | 2.77E-06 | TRUE | 1.46E-07 |
| rs7711427 | 1.01E-03 | 3.42E-07 | TRUE | 1.31E-10 |
| rs79045992 | 6.73E-04 | 4.83E-06 | TRUE | 1.02E-06 |
| rs79823250 | 6.70E-04 | 2.03E-07 | TRUE | 1.62E-07 |
| rs941823 | 1.14E-03 | 1.42E-05 | TRUE | 5.89E-10 |
| rs9611131 | 1.29E-03 | 4.48E-09 | TRUE | 1.46E-13 |
| rs9891119 | 9.47E-04 | 6.26E-07 | TRUE | 6.62E-10 |
| rs9911533 | 7.00E-04 | 2.78E-07 | TRUE | 9.31E-08 |
| rs9941524 | 1.22E-03 | 2.66E-05 | TRUE | 8.49E-10 |

**Supplemental Table S12.** **MR-steiger test results of the SNPs used in the Mendelian randomization analysis of the effects of inflammantary bowel disease on colorectal cancer risk**

| SNP | R^2^ of exposure | R^2^ of outcome | steiger direction | steiger p value |
| --- | --- | --- | --- | --- |
| rs10055349 | 1.18E-03 | 1.59E-05 | TRUE | 6.16E-13 |
| rs10142466 | 4.98E-04 | 2.37E-08 | TRUE | 1.61E-07 |
| rs10761659 | 3.57E-03 | 2.91E-07 | TRUE | 1.32E-44 |
| rs10800309 | 2.45E-03 | 5.95E-06 | TRUE | 8.23E-29 |
| rs11152949 | 1.47E-03 | 1.72E-07 | TRUE | 2.56E-19 |
| rs11190087 | 4.58E-04 | 9.95E-06 | TRUE | 1.61E-05 |
| rs11230563 | 8.96E-04 | 1.56E-08 | TRUE | 1.79E-12 |
| rs11641184 | 9.09E-04 | 2.80E-06 | TRUE | 1.62E-11 |
| rs11691685 | 6.46E-04 | 2.89E-06 | TRUE | 2.01E-08 |
| rs11713774 | 6.65E-04 | 3.15E-08 | TRUE | 1.40E-09 |
| rs12103 | 6.70E-04 | 3.39E-06 | TRUE | 1.30E-08 |
| rs12318183 | 1.80E-03 | 6.91E-07 | TRUE | 8.37E-23 |
| rs12411259 | 5.14E-04 | 8.94E-07 | TRUE | 2.74E-07 |
| rs12585310 | 6.56E-04 | 8.10E-08 | TRUE | 2.09E-09 |
| rs12599586 | 4.62E-04 | 2.15E-06 | TRUE | 2.14E-06 |
| rs12718244 | 8.75E-04 | 3.83E-06 | TRUE | 6.30E-11 |
| rs12720356 | 1.01E-03 | 2.08E-08 | TRUE | 7.84E-14 |
| rs12722515 | 7.29E-04 | 1.36E-05 | TRUE | 3.53E-08 |
| rs12796489 | 4.69E-03 | 2.83E-06 | TRUE | 2.06E-56 |
| rs12928665 | 4.93E-04 | 1.53E-07 | TRUE | 2.52E-07 |
| rs1297258 | 1.97E-03 | 1.72E-07 | TRUE | 2.47E-25 |
| rs13107612 | 6.91E-04 | 9.83E-06 | TRUE | 4.35E-08 |
| rs13204742 | 5.87E-04 | 4.11E-06 | TRUE | 1.53E-07 |
| rs1363907 | 9.33E-04 | 7.01E-06 | TRUE | 4.10E-11 |
| rs1388585 | 1.41E-03 | 8.46E-07 | TRUE | 4.89E-18 |
| rs1517352 | 8.71E-04 | 7.66E-06 | TRUE | 2.49E-10 |
| rs1528602 | 4.77E-04 | 1.33E-06 | TRUE | 9.99E-07 |
| rs1567009 | 1.08E-03 | 1.52E-06 | TRUE | 7.75E-14 |
| rs1569328 | 5.34E-04 | 3.78E-10 | TRUE | 4.77E-08 |
| rs17119 | 6.82E-04 | 1.67E-06 | TRUE | 4.32E-09 |
| rs17293632 | 1.30E-03 | 8.44E-06 | TRUE | 4.86E-15 |
| rs17668708 | 5.61E-04 | 1.65E-05 | TRUE | 3.50E-06 |
| rs17679361 | 5.14E-04 | 1.33E-06 | TRUE | 3.56E-07 |
| rs17694108 | 9.06E-04 | 6.08E-07 | TRUE | 4.05E-12 |
| rs17780256 | 6.71E-04 | 4.85E-06 | TRUE | 2.08E-08 |
| rs17800987 | 2.07E-03 | 2.08E-06 | TRUE | 1.75E-25 |
| rs181826 | 9.39E-04 | 7.31E-06 | TRUE | 3.89E-11 |
| rs1990623 | 9.79E-04 | 3.71E-06 | TRUE | 3.77E-12 |
| rs2024092 | 1.18E-03 | 1.09E-05 | TRUE | 1.85E-13 |
| rs2108225 | 6.98E-04 | 2.43E-06 | TRUE | 4.08E-09 |
| rs2143178 | 2.53E-03 | 1.24E-08 | TRUE | 1.68E-32 |
| rs2153283 | 6.93E-04 | 3.78E-06 | TRUE | 8.18E-09 |
| rs2270395 | 6.56E-04 | 3.23E-06 | TRUE | 1.75E-08 |
| rs2274351 | 5.11E-04 | 2.76E-09 | TRUE | 9.62E-08 |
| rs2284553 | 5.22E-04 | 1.18E-06 | TRUE | 2.67E-07 |
| rs2297559 | 6.86E-04 | 6.26E-06 | TRUE | 2.08E-08 |
| rs2393904 | 7.89E-04 | 9.45E-06 | TRUE | 3.26E-09 |
| rs2395022 | 9.17E-04 | 6.75E-07 | TRUE | 3.17E-12 |
| rs2488401 | 1.18E-03 | 1.35E-07 | TRUE | 9.79E-16 |
| rs2538470 | 6.73E-04 | 7.22E-07 | TRUE | 2.97E-09 |
| rs254562 | 5.80E-04 | 2.29E-05 | TRUE | 4.95E-06 |
| rs259964 | 7.16E-04 | 3.99E-10 | TRUE | 2.53E-10 |
| rs2688608 | 6.07E-04 | 1.58E-06 | TRUE | 3.23E-08 |
| rs272882 | 3.53E-03 | 1.77E-05 | TRUE | 4.29E-39 |
| rs2769267 | 5.26E-04 | 5.66E-06 | TRUE | 1.15E-06 |
| rs2816958 | 5.98E-04 | 3.95E-06 | TRUE | 1.06E-07 |
| rs2945412 | 4.70E-04 | 4.69E-06 | TRUE | 3.93E-06 |
| rs34779708 | 1.65E-03 | 1.42E-06 | TRUE | 1.05E-20 |
| rs34804116 | 4.62E-04 | 1.48E-07 | TRUE | 5.96E-07 |
| rs34856868 | 5.01E-04 | 1.59E-06 | TRUE | 5.90E-07 |
| rs35164067 | 1.30E-03 | 1.63E-06 | TRUE | 2.08E-16 |
| rs35256947 | 8.02E-04 | 1.08E-06 | TRUE | 1.09E-10 |
| rs3776414 | 8.82E-04 | 7.93E-07 | TRUE | 9.34E-12 |
| rs3801835 | 5.57E-04 | 1.15E-06 | TRUE | 9.93E-08 |
| rs3853824 | 5.76E-04 | 4.77E-06 | TRUE | 2.46E-07 |
| rs395157 | 1.30E-03 | 4.72E-06 | TRUE | 1.03E-15 |
| rs4643314 | 1.26E-03 | 6.46E-09 | TRUE | 6.01E-17 |
| rs4664304 | 4.72E-04 | 3.17E-06 | TRUE | 2.41E-06 |
| rs4692386 | 4.94E-04 | 1.00E-06 | TRUE | 5.11E-07 |
| rs4703855 | 6.47E-04 | 1.36E-08 | TRUE | 2.13E-09 |
| rs4743820 | 5.29E-04 | 1.58E-06 | TRUE | 2.73E-07 |
| rs4795397 | 2.93E-03 | 1.55E-05 | TRUE | 1.62E-32 |
| rs4845140 | 1.01E-03 | 3.74E-06 | TRUE | 1.86E-12 |
| rs4845604 | 1.11E-03 | 1.30E-06 | TRUE | 2.48E-14 |
| rs4917129 | 9.13E-04 | 1.97E-06 | TRUE | 9.34E-12 |
| rs4973341 | 6.16E-04 | 1.03E-05 | TRUE | 3.20E-07 |
| rs55693740 | 6.93E-04 | 6.29E-07 | TRUE | 1.57E-09 |
| rs55808324 | 1.07E-03 | 2.41E-06 | TRUE | 1.71E-13 |
| rs559928 | 8.07E-04 | 2.04E-07 | TRUE | 3.79E-11 |
| rs56163845 | 5.22E-04 | 1.41E-06 | TRUE | 2.97E-07 |
| rs56167332 | 3.35E-03 | 9.07E-10 | TRUE | 1.17E-42 |
| rs6025 | 4.73E-04 | 9.40E-09 | TRUE | 3.06E-07 |
| rs6074022 | 6.42E-04 | 6.66E-08 | TRUE | 2.98E-09 |
| rs6111031 | 4.86E-03 | 9.33E-08 | TRUE | 1.10E-60 |
| rs61959439 | 6.43E-04 | 7.85E-08 | TRUE | 3.02E-09 |
| rs6456426 | 6.43E-04 | 9.16E-06 | TRUE | 1.29E-07 |
| rs648541 | 5.62E-04 | 2.01E-05 | TRUE | 5.39E-06 |
| rs6561151 | 1.08E-03 | 1.64E-06 | TRUE | 7.77E-14 |
| rs6588248 | 1.04E-03 | 7.07E-09 | TRUE | 2.78E-14 |
| rs6651252 | 5.71E-04 | 7.41E-07 | TRUE | 5.06E-08 |
| rs6740462 | 7.23E-04 | 9.27E-07 | TRUE | 8.75E-10 |
| rs67643815 | 5.81E-04 | 1.43E-09 | TRUE | 1.24E-08 |
| rs7011507 | 4.79E-04 | 3.50E-07 | TRUE | 4.73E-07 |
| rs7015630 | 4.69E-04 | 3.13E-06 | TRUE | 2.58E-06 |
| rs7097656 | 9.74E-04 | 1.85E-06 | TRUE | 1.68E-12 |
| rs7194886 | 2.41E-03 | 3.05E-06 | TRUE | 4.13E-29 |
| rs7236492 | 4.89E-04 | 2.68E-08 | TRUE | 2.09E-07 |
| rs7240004 | 6.36E-04 | 6.26E-07 | TRUE | 7.51E-09 |
| rs72634258 | 1.25E-03 | 2.10E-09 | TRUE | 6.58E-17 |
| rs72924296 | 4.89E-04 | 8.47E-07 | TRUE | 5.34E-07 |
| rs72978783 | 5.30E-04 | 2.87E-06 | TRUE | 4.59E-07 |
| rs7404095 | 4.67E-04 | 1.15E-06 | TRUE | 1.20E-06 |
| rs752508 | 6.05E-04 | 1.72E-06 | TRUE | 3.60E-08 |
| rs7711427 | 4.47E-03 | 3.42E-07 | TRUE | 1.62E-55 |
| rs7773324 | 5.16E-04 | 1.40E-06 | TRUE | 3.53E-07 |
| rs7848647 | 2.33E-03 | 4.11E-06 | TRUE | 6.85E-28 |
| rs8055876 | 6.16E-04 | 3.80E-06 | TRUE | 6.37E-08 |
| rs8127691 | 1.95E-03 | 1.77E-06 | TRUE | 3.57E-24 |
| rs925255 | 8.82E-04 | 4.40E-08 | TRUE | 3.06E-12 |
| rs941823 | 7.88E-04 | 1.42E-05 | TRUE | 8.87E-09 |
| rs9457247 | 1.16E-03 | 1.55E-09 | TRUE | 8.31E-16 |
| rs9557207 | 8.05E-04 | 1.86E-06 | TRUE | 1.66E-10 |
| rs9889296 | 1.32E-03 | 1.37E-08 | TRUE | 1.15E-17 |

**Supplemental Table S13.** **MR-steiger test results of the SNPs used in the Mendelian randomization analysis of the effects of colorectal cancer on crohn’s disease risk**

| SNP | R^2^ of exposure | R^2^ of outcome | steiger direction | steiger p value |
| --- | --- | --- | --- | --- |
| rs16892766 | 9.28E-05 | 2.38E-05 | TRUE | 3.10E-01 |
| rs3087967 | 1.22E-04 | 7.87E-05 | TRUE | 6.42E-01 |
| rs4939827 | 1.88E-04 | 2.00E-08 | TRUE | 3.76E-03 |
| rs6983267 | 2.43E-04 | 7.05E-06 | TRUE | 5.73E-03 |

**Supplemental Table S14.** **MR-steiger test results of the SNPs used in the Mendelian randomization analysis of the effects of colorectal cancer on ulcerative colitis risk**

| SNP | R^2^ of exposure | R^2^ of outcome | steiger_dir | steiger p value |
| --- | --- | --- | --- | --- |
| rs16892766 | 9.28E-05 | 1.67E-06 | TRUE | 8.60E-02 |
| rs3087967 | 1.22E-04 | 9.44E-05 | TRUE | 7.84E-01 |
| rs4939827 | 1.88E-04 | 4.52E-06 | TRUE | 1.71E-02 |
| rs6983267 | 2.43E-04 | 3.57E-05 | TRUE | 4.77E-02 |

**Supplemental Table S15.** **MR-steiger test results of the SNPs used in the Mendelian randomization analysis of the effects of colorectal cancer on inflammantary bowel disease risk**

| SNP | R^2^ of exposure | R^2^ of outcome | steiger direction | steiger p value |
| --- | --- | --- | --- | --- |
| rs16892766 | 9.28E-05 | 6.58E-06 | TRUE | 9.46E-02 |
| rs3087967 | 1.22E-04 | 8.08E-05 | TRUE | 6.26E-01 |
| rs4939827 | 1.88E-04 | 7.48E-06 | TRUE | 9.46E-03 |
| rs6983267 | 2.43E-04 | 3.27E-05 | TRUE | 1.95E-02 |

**Supplemental Figure S1.“Leave-one-out” sensitivity analysis of causal effects of crohn’s disease on colorectal cancer.**


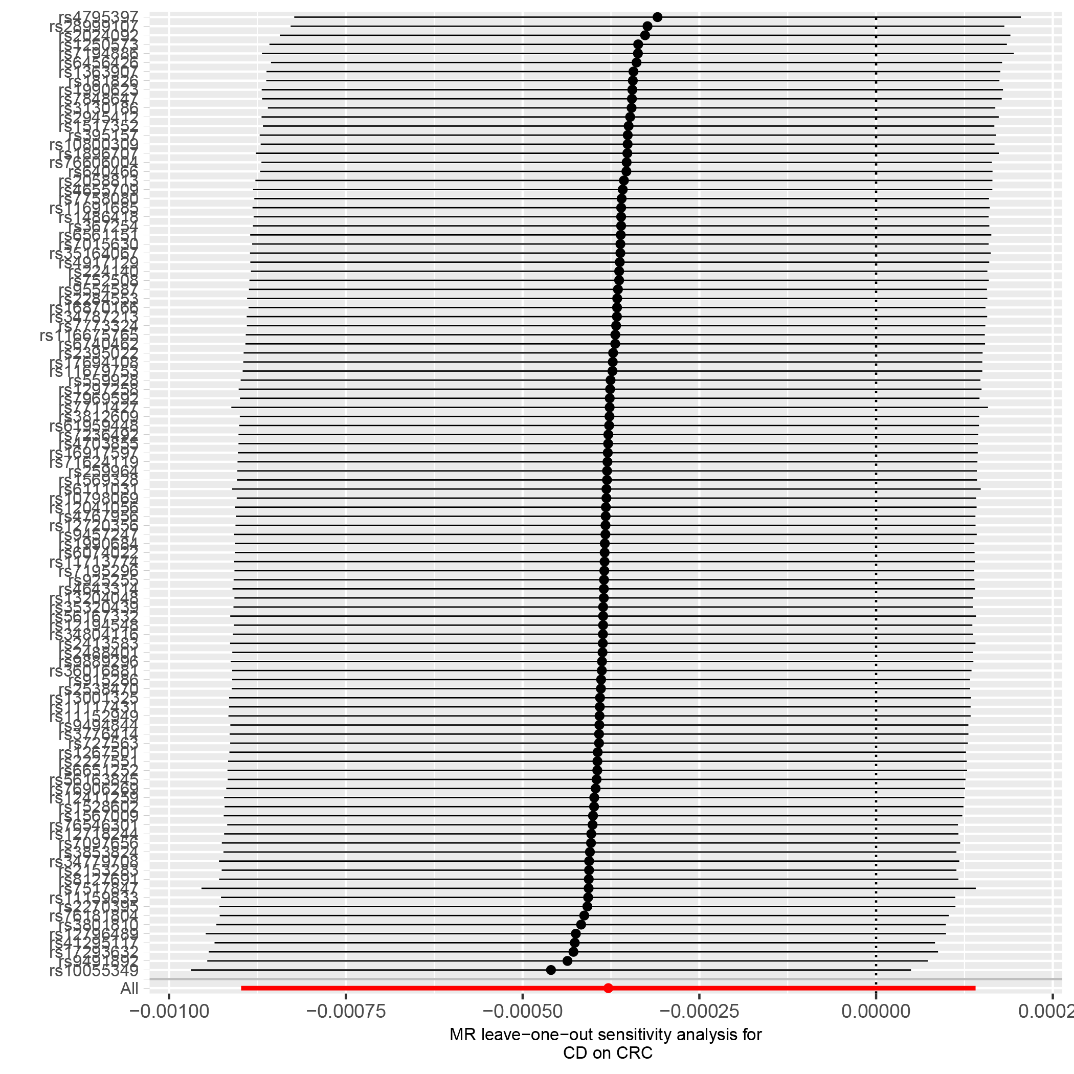


Note: The significance of the red line is the MR results of the inverse variance weighting (IVW) method.

**Supplemental Figure S2. “Leave-one-out” sensitivity analysis of causal effects of ulcerative colitis on colorectal cancer.**


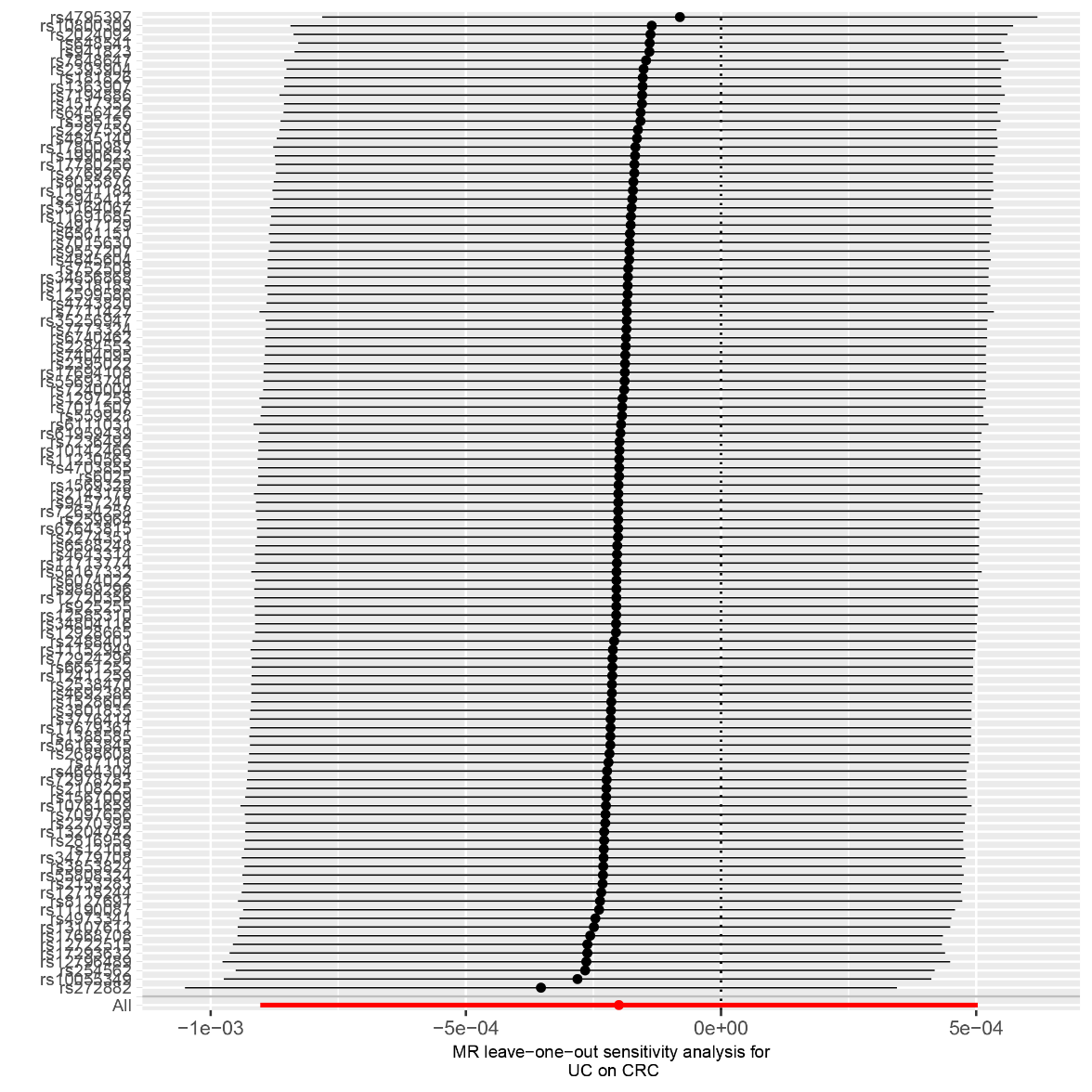


Note: The significance of the red line is the MR results of the inverse variance weighting (IVW) method.

**Supplemental Figure S3. “Leave-one-out” sensitivity analysis of causal effects of inflammatory bowel disease on colorectal cancer.**


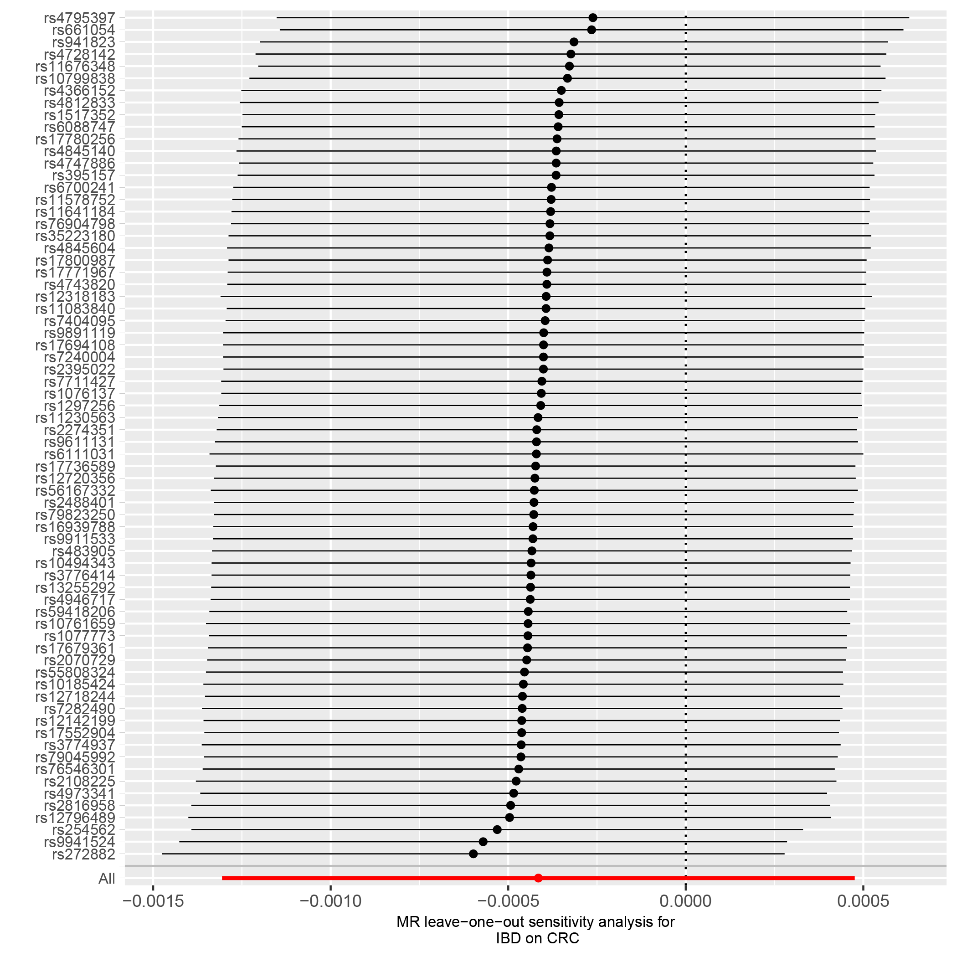


Note: The significance of the red line is the MR results of the inverse variance weighting (IVW) method.

**Supplemental Figure S4. “Leave-one-out” sensitivity analysis of causal effects of colorectal on crohn’s disease.**


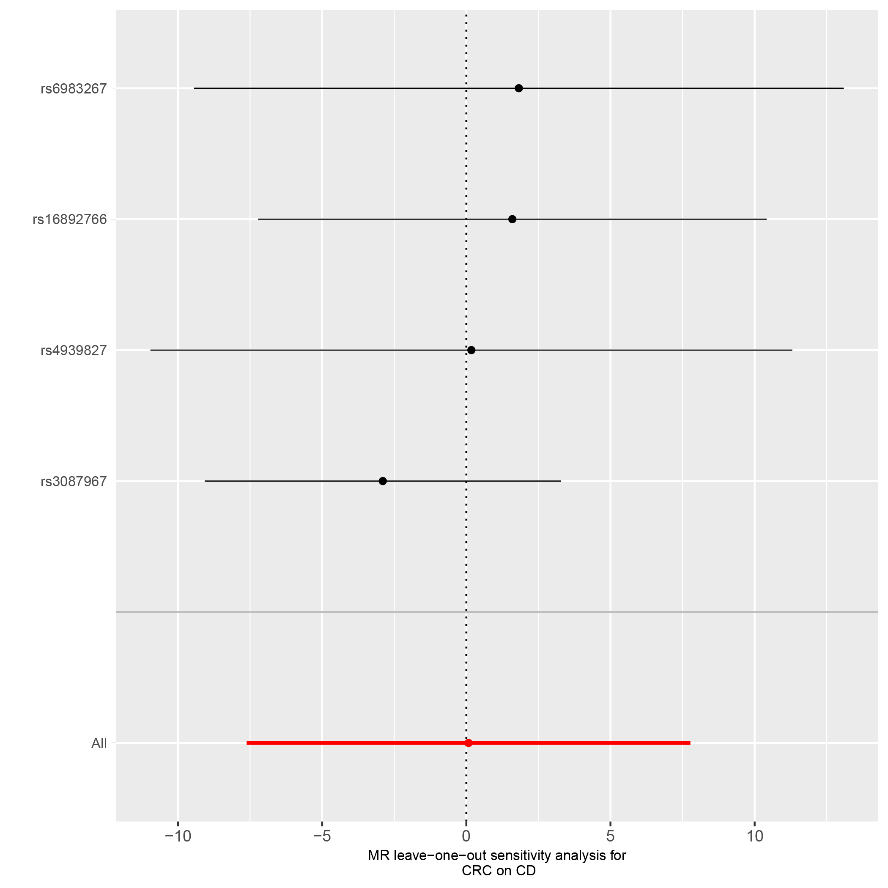


Note: The significance of the red line is the MR results of the inverse variance weighting (IVW) method.

**Supplemental Figure S5. “Leave-one-out” sensitivity analysis of causal effects of colorectal cancer on ulcerative colitis.**


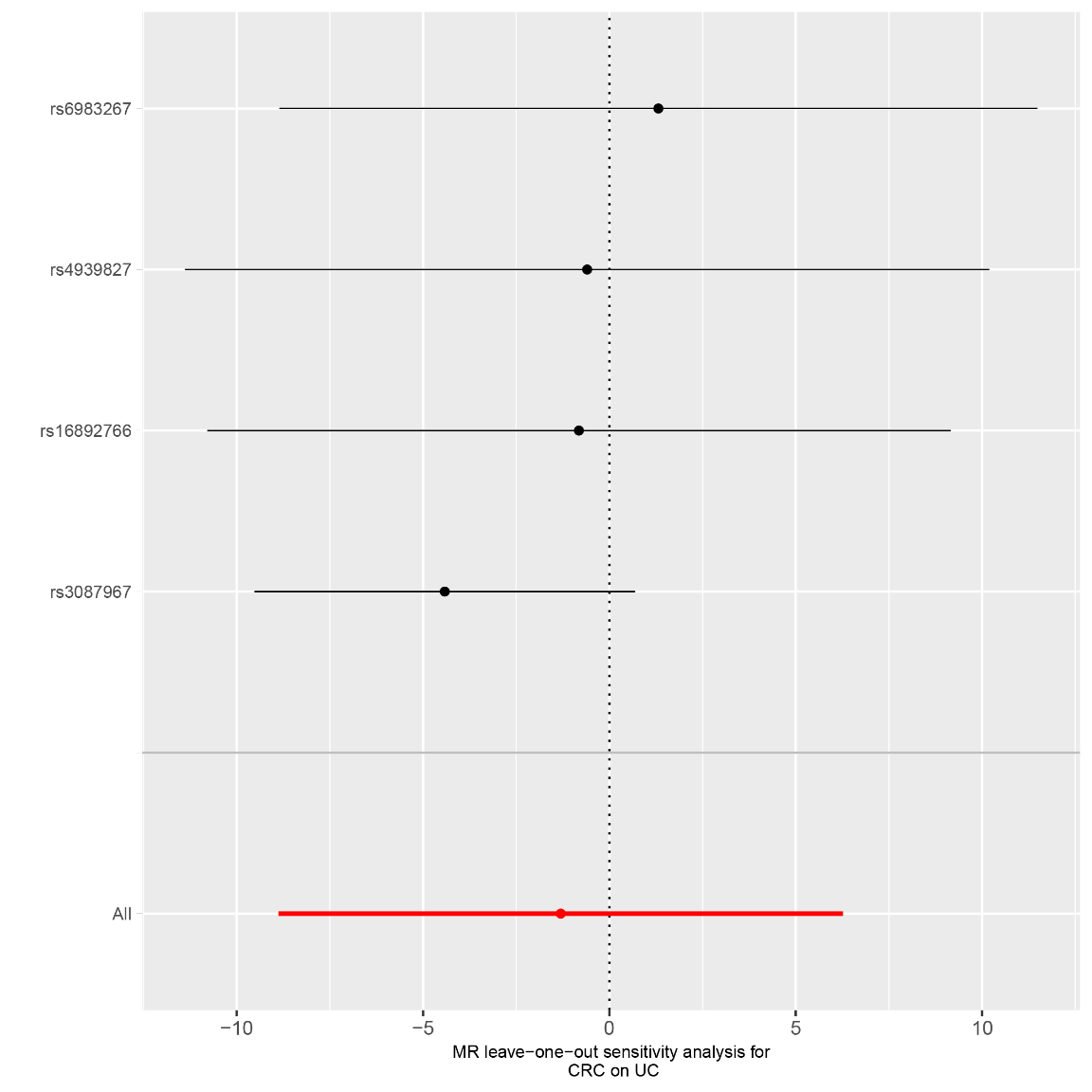


Note: The significance of the red line is the MR results of the inverse variance weighting (IVW) method.

**Supplemental Figure S6. “Leave-one-out” sensitivity analysis of causal effects of colorectal cancer on inflammatory bowel disease.**


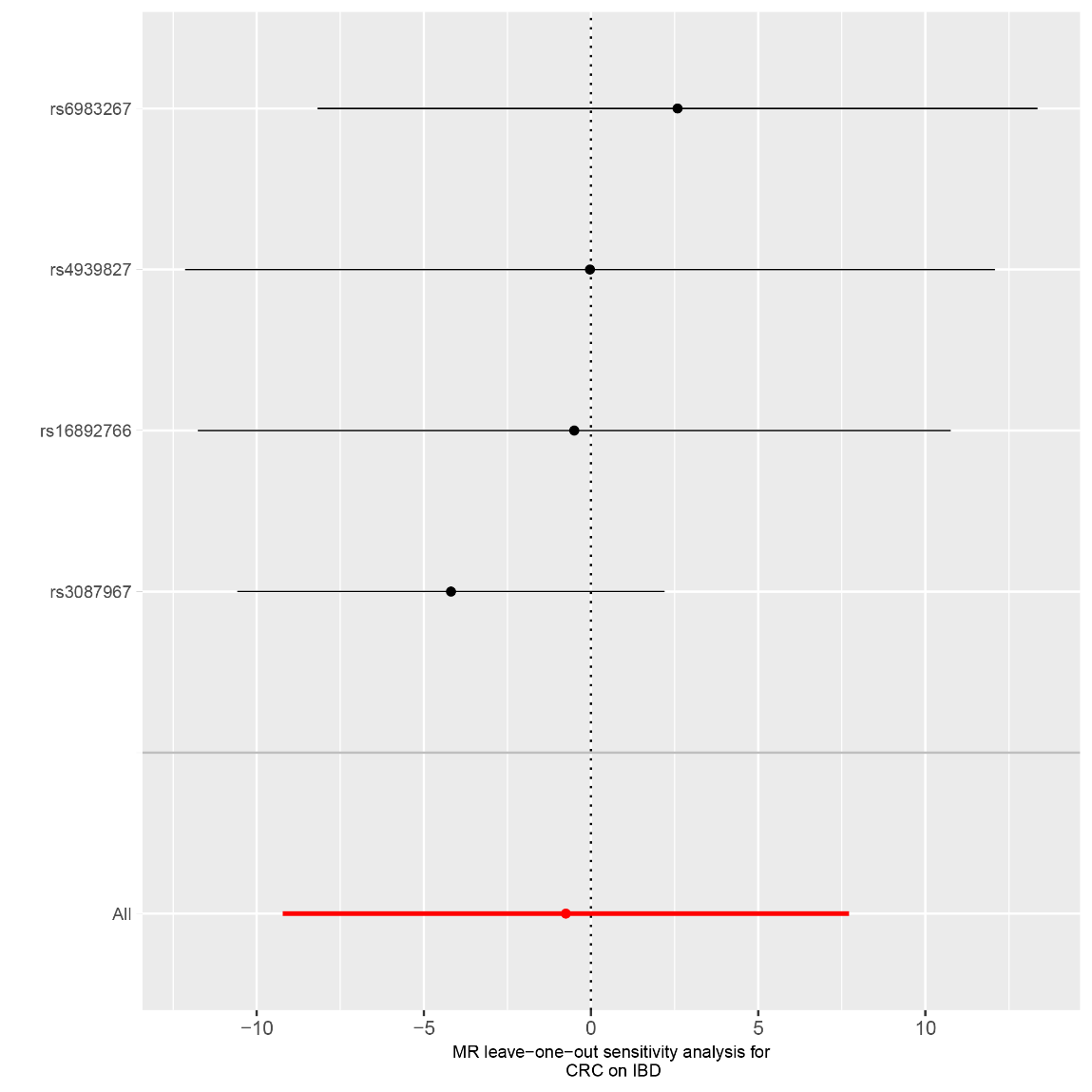


Note: The significance of the red line is the MR results of the inverse variance weighting (IVW) method.

**Supplemental Figure S7. Forest plot of the Mendelian randomization (MR) outcome.**


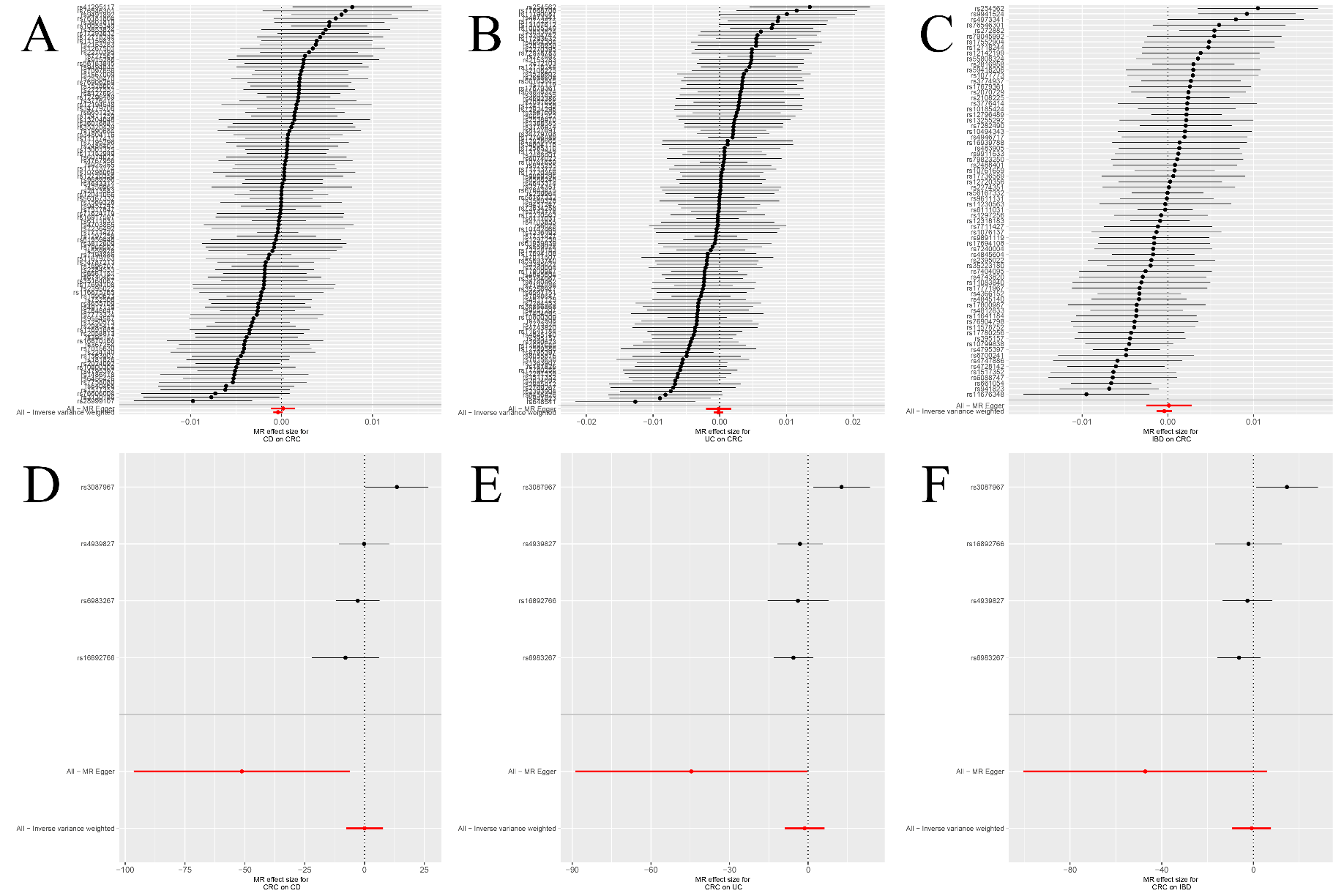


(A) Effect of CD on CRC, (B) effect of UC on CRC, (C) effect of IBD on CRC, (D) effect of CRC on CD, (E) effect of CRC on UC, (F) effect of CRC on IBD.

**Supplemental Figure S8. Funnel plot of genetic associations with exposure against causal estimates based on each genetic variant individualy.**


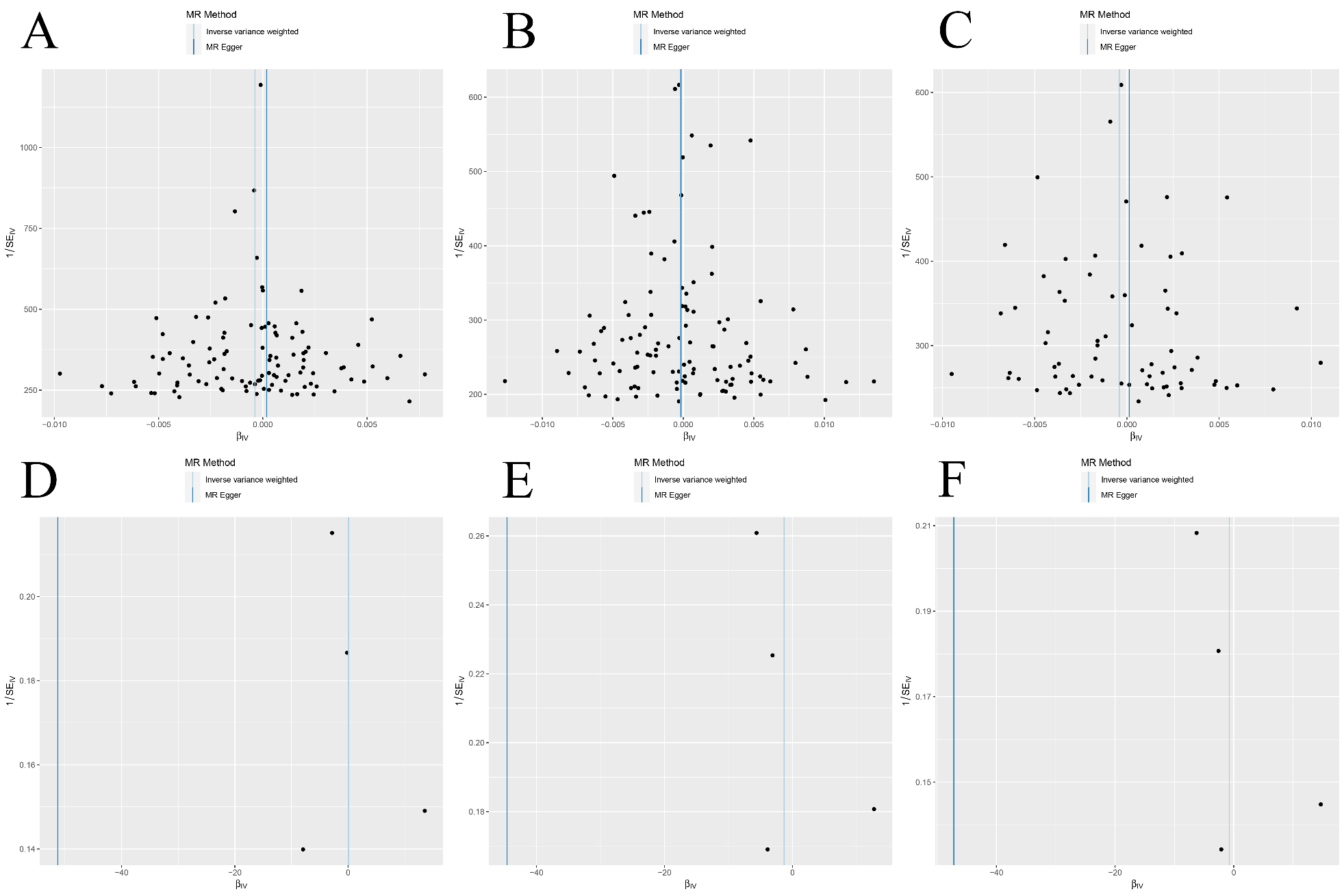


(A) Effect of CD on CRC, (B) effect of UC on CRC, (C) effect of IBD on CRC, (D) effect of CRC on CD, (E) effect of CRC on UC, (F) effect of CRC on IBD.
